# MuSK Myasthenia Gravis IgG4 Disrupts the Interaction of LRP4 with MuSK but Both IgG4 and IgG1-3 Can Disperse Preformed Agrin-Independent AChR Clusters

**DOI:** 10.1371/journal.pone.0080695

**Published:** 2013-11-07

**Authors:** Inga Koneczny, Judith Cossins, Patrick Waters, David Beeson, Angela Vincent

**Affiliations:** Neurosciences Group, Nuffield Department of Clinical Neurosciences, Weatherall Institute of Molecular Medicine, University of Oxford, Oxford, United Kingdom; Georgia Regents University, United States of America

## Abstract

A variable proportion of patients with generalized myasthenia gravis (MG) have autoantibodies to muscle specific tyrosine kinase (MuSK). During development agrin, released from the motor nerve, interacts with low density lipoprotein receptor-related protein-4 (LRP4), which then binds to MuSK; MuSK interaction with the intracellular protein Dok7 results in clustering of the acetylcholine receptors (AChRs) on the postsynaptic membrane. In mature muscle, MuSK helps maintain the high density of AChRs at the neuromuscular junction. MuSK antibodies are mainly IgG4 subclass, which does not activate complement and can be monovalent, thus it is not clear how the antibodies cause disruption of AChR numbers or function to cause MG. We hypothesised that MuSK antibodies either reduce surface MuSK expression and/or inhibit the interaction with LRP4. We prepared MuSK IgG, monovalent Fab fragments, IgG1-3 and IgG4 fractions from MuSK-MG plasmas. We asked whether the antibodies caused endocytosis of MuSK in MuSK-transfected cells or if they inhibited binding of LRP4 to MuSK in co-immunoprecipitation experiments. In parallel, we investigated their ability to reduce AChR clusters in C2C12 myotubes induced by a) agrin, reflecting neuromuscular development, and b) by Dok7- overexpression, producing AChR clusters that more closely resemble the adult neuromuscular synapse. Total IgG, IgG4 or IgG1-3 MuSK antibodies were not endocytosed unless cross-linked by divalent anti-human IgG. MuSK IgG, Fab fragments and IgG4 inhibited the binding of LRP4 to MuSK and reduced agrin-induced AChR clustering in C2C12 cells. By contrast, IgG1-3 antibodies did not inhibit LRP4-MuSK binding but, surprisingly, did inhibit agrin-induced clustering. Moreover, both IgG4 and IgG1-3 preparations dispersed agrin-independent AChR clusters in Dok7-overexpressing C2C12 cells. Thus interference by IgG4 antibodies of the LRP4-MuSK interaction will be one pathogenic mechanism of MuSK antibodies, but IgG1-3 MuSK antibodies will also contribute to the reduced AChR density and neuromuscular dysfunction in myasthenia patients with MuSK antibodies.

## Introduction

Myasthenia gravis (MG) is a rare but often severe disorder of neuromuscular transmission that causes fatigable muscle weakness. The majority of patients have antibodies to the acetylcholine receptor (AChR; reviewed by Punga and Ruegg [1] and Spillane [2]). A proportion of those patients who do not have AChR antibodies have, instead, antibodies to muscle-specific tyrosine kinase (MuSK) [3], and a small number of patients have antibodies to lipoprotein receptor-related protein-4 (LRP4) [4,5], another neuromuscular junction membrane protein which interacts with MuSK. 

 MuSK is a key organizer in postsynaptic development, when it orchestrates the clustering of the AChR beneath the motor nerve presynaptic bouton [6]. Agrin is released by the motor neuron [7] and binds to LRP4 which then binds to and activates MuSK. This results in MuSK autophosphorylation and down-stream signalling, via the cytoplasmic adaptor-like protein Dok7 [8,9] and the AChR associated protein rapsyn [6,10,11], leading to AChR clustering (reviewed in 12,13). MuSK antibodies were found to interfere with this signalling pathway *in vitro*, reducing agrin-induced AChR clustering in C2C12 myotubes [3].

In MG patients with AChR antibodies, IgG1 and IgG3 antibodies bind to the AChR and activate complement, cause internalisation of the AChRs, and/or block AChR function; the overall effect is to cause loss of AChRs or reduction in their function. If MuSK antibodies similarly caused loss of MuSK or inhibited MuSK function, one would expect reduced AChR numbers or stability of the AChR clusters; indeed most studies of MuSK immunised animals have found reduced AChR numbers [14] and disruption of the AChR clusters on the postsynaptic membrane [15,16]. Similar observations have been made after injection of MuSK MG patients’ IgG antibodies [14,17-19], and in one study IgG4 but not IgG1-3 was implicated [19]. However, how MuSK antibodies act is not clear, and although one publication suggested endocytosis of MuSK as a mechanism [20] this would be unexpected for IgG4 antibodies that are thought to be largely monovalent. Indeed, we did not find evidence for internalisation of MuSK by purified IgGs *in vitro*, but MuSK IgG, MuSK Fab fragments and IgG4, although not MuSK IgG1-3, blocked binding of LRP4 to MuSK. Surprisingly, however, MuSK IgG1-3 did impair agrin-induced AChR clustering in C2C12 myotubes, and both IgG1-3 and IgG4 MuSK antibodies dispersed preformed clusters in agrin-independent Dok7-overexpressing C2C12 cells. These results add new information to the complex mechanisms by which IgG1-3 and IgG4 binding to MuSK cause MuSK antibody-induced myasthenia.

## Methods

### Patients

We used plasma from patients identified as having MuSK antibodies [3] and described briefly in the Table 1.

**Table 1:** Clinical data of 14 MuSK-MG patients.

**Patient designation**	**MuSK Antibody titre (nM) in plasma**	**Age of onset**	**Sex** ^a^	**Duration at sample day (years)**	**MGFA at day of sample**	**Max MGFA**	**Predominan involvement^b^**	**Treatment ^c^**	**Response to treatment**	**Outcome (clinical evaluation at last FU) ^d^**
1	37.57	17	F	1	IVB	IVB	OBR	Py,Pr,Az,PX,TE	+(except TE)	R
2	32.46	5	F	na	IVB	IVB	OR	Pr,Az,Mt,IVIG,PX	+++	+I,S
3	25.65	42	M	na	IIIB	IIIB	na	PX, na	+	na
4	19.37	23	F	19	IIIB	V	BR	Py,Pr,Az,Cy,IVIG,PX,TE	+/(IS),+(PX),-(TE)	I,S (T)
5	18.87	23	F	11	IIIB	V	BR	Py,Pr,PX	++(Pr,PX)	R
6	14.48	3	F	5	IIIB	IIIB	FB	Py,Pr,Az,Cy,Ri,PX	+++(Ri,PX)	R (T)
7	14.7	27	F	5	IIIB	IIIB	FB	Py,Pr,Az,PX	+(IS,PX)	R,(T)
8	10.72	18	F	<1	IVB	IVB	B	Pr,Az,PX	++	++I
9	10.51	14	F	<1	IIIB	IIIB	B	Py,Pr,Az,PX	+	++I
10	10.47	25	F	2	IIIB	IVB	L	Pr,Az,Mt,My,PX	+	Sy (T)
11	7.1	28	F	<1	IIB	IIB	OB	PX, na	na	na
12	6.48	48	F	<1	IIB	V	B	PX, na	++	na
13	4.02	28	M	2	IIIB	IIIB	OF	Py,Pr,Az,PX	+(IS)	R
14	1.03	17	F	1	IVB	IVB	FB	Pr,Az,PX	+	++I,Sy

^a^ F=female, M=male; ^b^ O=ocular, R=respiratory, F=facial, B=bulbar, L=limbs; ^c^ Pr=Prednisolone, Az=Azathioprine, Mt=Methotrexate, IVIG=intravenous IG, PX=plasma exchange, Cy=cyclosporine, Ri=Rituximab, TE=thymectomy, My=Mycophenalete, IS=immunosuppression; ^d^ I=improvement, R=remission, (T)=still under treatment, Sy=still showing symptoms, na=data not available, + good,++ very good, +++ remarkably good. Some plasmas were sent by colleagues elsewhere, or were from patients who returned to their countries, and follow-up data are not available.

### Ethics statement

The MG samples were archived from therapeutic plasmaphereses in the 1980s and 1990s when written consent was not required, although patients gave verbal consent for storage and research use of the plasmas. Ethical approval for use of pre 2006 stored patient samples without patient written consent was obtained from the Oxfordshire REC C 09/H0606/74. Samples from healthy individuals were obtained with written consent and ethical approval from the Oxfordshire REC Rf 07/Q1604/28.

### Tissue culture

Human embryonic kidney 293 (HEK293, derived from ECACC) cells were maintained in HEK cell growth medium (DMEM (Sigma, D6429) supplemented with 10% foetal calf serum (PAA, A15-151) and 1% antibiotic-antimycotic (Gibco, 15240)) at 37°C, 5.5% CO_2_ at 30-80% confluency. C2C12 mouse myoblasts (derived from ECACC) were maintained in growth medium (DMEM supplemented with 15% Foetal calf serum and 1% antibiotic-antimycotic) and differentiated to form myotubes for 4-7 days in differentiation medium (DMEM supplemented with 2% Foetal calf serum and 1% antibiotic-antimycotic) at 37°C, 5.5% CO_2_.

### Radioimmunoprecipitation assays (RIA)

MuSK and AChR antibody titres were determined by RIA as previously described [21]. Plasmas were serially diluted 5-fold in PTX (0.02M PBS (Oxoid, BK0014G), 0.1% Triton X) to a total volume of 50 µl and incubated with 50µl of ^125^I labelled AChR or MuSK (RSR Ltd.) and 5µl carrier serum (healthy control serum) overnight at 4°C. 50µl anti-human IgG (RSR Ltd.) was added and incubated at room temperature until precipitation was visible (approximately 60 minutes). After addition of 500µl PTX for washing, the samples were centrifuged 5 min 13000g at room temperature. The pellet was washed twice with 500µl PTX, and the cpm were measured with a Wallac Wizard counter.

### Flow cytometry analysis of IgG subclasses

HEK293 cells were transfected with DNA encoding either full length human MuSK inserted into the pIRES2-EGFP vector (Clontech), the empty vector or no DNA using polyethyleneimine. 1x10^5^ cells were incubated with a 1:50 dilution of patient plasma or healthy control serum in staining medium (DMEM supplemented with 1% Anti-anti, 1% BSA (Sigma-Aldrich, A7906-100G), 20mM HEPES (Sigma, H3375-100G)) containing 10% goat serum (Sigma Aldrich, G9023) for 20 minutes at 4°C, with shaking. After washing the cells in 500µl staining medium, cells were fixed for 5 minutes in 1% formaldehyde (TAAB Laboratories Equipment Ltd, F003) in staining medium at 4°C, washed, and incubated with a 1:50 dilution of mouse anti-human IgG1, 2, 3 or 4 (The Binding Site, MC003,11,6,5) in staining medium for 30 minutes at 4°C, with shaking. After washing the cells with staining medium, they were incubated with 1:200 APC-conjugated anti-mouse antibody (Invitrogen, A21235) in staining medium for 30 minutes at 4°C in the dark. Cells were washed and resuspended in PBS supplemented with 2.5mM EDTA, pH8.0 (Gibco, 15575) before analysis with a CyanADP flow cytometer. GFP positive cells were gated and analysed for median far red fluorescence intensity.

### IgG purification

10 ml plasma was diluted 1:2 in Hartmann's solution (compound sodium lactate, Baxter healthcare, FKE2323) and incubated with 2.5 ml protein G sepharose (Sigma, P3296) over night at 4°C with gentle shaking. Bead slurry was loaded onto a column, washed with 50ml Hartmann's solution, and bound IgG was eluted with 0.1M glycine, pH 2.3. 20 x 1ml fractions were collected, neutralised and analysed for protein content by measuring the OD280. Fractions with OD2801 were pooled and dialysed over night against 200 volumes of Hartmann's solution. 

### Fab purification

Purified IgGs were filtered (0.20um syringe filter, Sartorius, 16534), desalted using 5ml Zeba Spin desalting columns (Thermo scientific, 89892) and diluted to a maximum protein concentration of 4mg/ml in Hartmann's solution. Papain beads (Thermo scientific, 20341) were pre-washed in digestion buffer from the Pierce Fab preparation kit (Thermo scientific, 44985), and then incubated with two-fold volume of the purified IgGs over night at 37°C with gentle shaking. The digest was centrifuged for 1 minute at 5000g and the supernatant containing Fab fragments was transferred to a fresh tube, and incubated for 1h at 4°C with 1ml protein G sepharose (Sigma, P3296). Flow-through and wash containing purified Fab fragments were pooled, and the Fc fragments and undigested IgG were eluted with 0.1M glycine pH 2.3. Aliquots from all steps were analysed by gel electrophoresis with non-reducing conditions and coomassie-staining using PageBlue Protein Staining solution (Thermo scientific, 24620). The concentrations of Fabs and whole IgG were verified by RIA using immunoprecipitation by the polyspecific anti-human IgG (RSR Ltd) that binds to Fab as well as to Fc IgG fragments (confirmed by preliminary experiments). The preparations were applied at defined MuSK-antibody concentrations based on these results.

### IgG4 and IgG1-3 purification

10ml patient plasma was filtered and diluted 1:2 with PBS, then incubated for 1h at 4°C with 1ml IgG4 affinity matrix (Captureselect, 2900.05) and packed into a column. After washing with 3 x 10ml PBS, IgG4 was eluted with 0.1M glycine pH 2.3 in 20 x 1ml fractions. The 4 fractions with the highest OD280 were pooled and dialysed over night against 200 volumes of Hartmann's solution. The flow-through containing IgG1-3 was loaded a second time onto the IgG4 affinity matrix to ensure complete depletion of IgG4, and then incubated over night with 2.5ml protein G sepharose and eluted as described for IgG purification. Purity of the subclasses was verified using subclass-specific flow cytometry, and MuSK antibody titres were measured with the RIA. All preparations were applied at defined MuSK-antibody concentrations based on these results.

### Production of agrin

A T175 flask with HEK293 at approximately 60% confluency were transfected with 42μg of a construct expressing a soluble c-terminal splice form of neural agrin (donated by the late Dr.Werner Hoch, for details see 22) using 42 µl polyethyleneimine and 35µl 20% glucose. After 24 hours, the medium was replaced with HEK cell growth medium. 48 hours later the conditioned medium was centrifuged at 1200g for 10 minutes at room temperature, aliquoted and stored at -20°C.

### Endocytosis study

HEK293 cells were seeded at 3x10^5^ cells per well onto poly-L lysine-coated glass coverslips in a 6-well plate, and were transfected the following day with 4µg of full length human MuSK inserted into the pIRES2-EGFP vector using polyethyleneimine. 40 hours after transfection, the coverslips were transferred into 24 well plates. Half of the coverslips were fixed for 10 minutes with 3% formaldehyde prior to incubation with the primary antibody to prevent endocytosis. These will be referred to as “pre-fixed”. The other half was unfixed. Samples containing patient plasma, purified IgG, IgG4 or IgG1-3 were diluted in staining medium to a final MuSK antibody titre of 0.17nM; this was limited by the low concentration of MuSK IgG1-3 in the plasma. The samples were blinded, and then applied to the coverslips with living or pre-fixed cells and incubated with gentle rocking for 30 minutes at 4°C. To obtain first the binding of the antibodies without endocytosis we used 30 minutes incubation at 4°C. The supernatant was then aspirated and cells were washed 3 times with HEK cell growth medium. In experiments with anti-human IgG cross-linking, the anti-human IgG Alexa Fluor 568 (Invitrogen, A21090) diluted 1:200 in staining medium, was added at this stage, incubated for 30 minutes at 4°C, with gentle rocking, and washed 3 times with HEK cell growth medium, before moving the plates to either 4°C (to limit endocytosis) or 37°C (at which endocytosis can occur) and incubated for up to 16 hours. At different time points, the cells were fixed for 5 minutes with 3% formaldehyde. Bound human IgG was identified by staining with anti-human IgG Alexa Fluor 568 (Invitrogen, A21090) diluted 1:750 in staining medium for 45 minutes at RT in the dark. This step was usually omitted in the cross-linking experiments as secondary antibody had already been added). Coverslips were washed with PBS, and then the 24-well plates were blinded prior to analysis. Red fluorescence was analyzed and the fluorescence intensity of the samples was scored blinded by two independent individuals, with scores from 0=no antibody binding to 4=strong antibody binding. Scores at time point 0hr for each sample were set as 100% and scores at subsequent time points were normalized to this. 

### AChR clustering assay

C2C12 myotubes were incubated for 16 hours with or without soluble rat agrin diluted 1:100 in differentiation medium and with purified IgG or Fab fragments from MuSK MG-patients diluted to the same titre, or with the same volumes of healthy control IgG or Fab fragments. Samples were blinded prior to application. AChR clusters were stained with Alexa Fluor 594-conjugated α-bungarotoxin (Invitrogen, B13423) diluted 1:1000 in differentiation medium to a final concentration of 1µg/ml for 60 minutes at 37°C, 5.5% CO_2_, washed with differentiation medium 3 x 5 minutes and fixed for 20 minutes with 3% formaldehyde at room temperature in the dark. Myotubes were washed with PBS and stored in PBS at 4°C. 30 fields containing myotubes visualised in the bright field were selected and microscopic images of red fluorescence were acquired using Simple PCI (Digital Pixel) and analysed for AChR cluster number and length using ImageJ software. 

### Co-immunoprecipitation

HEK293 cells were seeded at 7.5x10^4^ cells per well in 24-well plates. They were transfected the following day with mammalian expression vectors expressing full-length human MuSK with mCherry fused to the C-terminus, or full-length human LRP4 with EGFP fused to the C-terminus, or both. 48 hours later, each well was incubated with 40µl anti MuSK antibody (R&D Systems, polyclonal goat, AF562) in 250µl HEK growth medium, or MuSK-MG plasma diluted in HEK growth medium to a final concentration of 1nM MuSK antibodies, for 2h at 37°C, 5.5% CO_2_. Cells were washed 3 times in growth medium, resuspended in 220µl ice-cold lysis buffer (10mM Tris-HCl, pH 7.4, 100mM NaCl, 1mM EDTA, 1%Triton-X supplemented with 1:100 protease inhibitor cocktail (Sigma)) and placed on ice for 15 minutes. Cell lysate was centrifuged 15 min 12000g at 4°C. A 10µl aliquot of the supernatant was reserved as whole cell lysate control. 200µl of the supernatant was incubated with 25µl protein G coupled magnetic beads (Invitrogen, Dynabeads, 100.03D) for 20 minutes at room temperature with gentle agitation. The beads were washed 6 times with 200µl lysis buffer, resuspended in 15µl of NuPage LDS sample buffer (Invitrogen, NP0008) containing NuPage reducing agent (Invitrogen, NP0004), and heated for 10 minutes at 70°C. Protein was separated by SDS PAGE using NuPage Novex Tris-acetate mini gels (Invitrogen) and transferred to nitrocellulose. HiMark Pre-stained High molecular weight protein standard, (Invitrogen, LC5699) was also included. MuSK-mCherry was detected using 1:1000 anti-mCherry polyclonal antibody (BioVision, 5993-100), and LRP4-EGFP was detected with 1:2000 anti-GFP antibody (Abcam ab6556), each diluted in PBS supplemented with 0.1% Tween and 1% milk powder. HRP-conjugated secondary antibodies were from Dako and were detected using ECL from GE Healthcare.

For each patient or healthy control sample, the area density of each band corresponding to MuSK on the western blot was measured with VisionWorks®LS Image Acquisition Software (UVP 97-0186-03). Area density of the patient and healthy control samples was normalized to the area density of the MuSK band immuno-precipitated by the commercial anti-MuSK antibody. Likewise the area densities of each LRP4 band pulled down by patient and control samples were measured and normalised to the area density of the LRP4 band brought down by the commercial anti-MuSK antibody. Since there was some variation in the amount of MuSK pulled down by each patient, a value called “relative LRP4-MuSK binding efficiency” was calculated as the ratio between the normalised area densities of the LRP4 and MuSK bands.

### Generation of C2C12 myotubes overexpressing Dok7

C2C12 myotubes overexpressing Dok7 were prepared as described previously (Cossins et al., 2012). Briefly, a pBABE-IRES-EGFP retrovirus containing the cDNA for human Dok7 was used to infect C2C12 myoblasts. Infected cells were selected using puromycin. Expression of EGFP was a marker of successful infection. After differentiation AChR clusters were visualised using Alexa Fluor 594-conjugated α-bungarotoxin. 

### Statistics

All data were analyzed with GraphPad Prism 6. The relationships between MuSK antibody titres and IgG subclasses were analysed by Pearson regression. For AChR clustering and Co-IP, data from at least 3 experiments for each patient preparation were pooled and analyzed with one way ANOVA using Bonferroni post-test comparing all columns against a control column. All error bars represent standard error of the mean. 

## Results

### The MuSK-antibody patients

14 MuSK patients (12 female, 2 male) were studied (Table 1). The ages at onset were 3-48 years, and all had generalized symptoms. Maximum MGFA scores were IIB-V, and MGFA scores preceding plasma exchange were between IIB and IVB. All patients with full data available had received other treatments, usually prednisolone and azathioprine, and most had also received IVIG, rituximab or methotrexate at some stage in their illness. Plasmas or sera from thirty healthy individuals aged 23-70 years and five AChR-MG patients were used for comparisons. 

### MuSK antibodies are mainly of the IgG4 subclass

**Figure 1:**
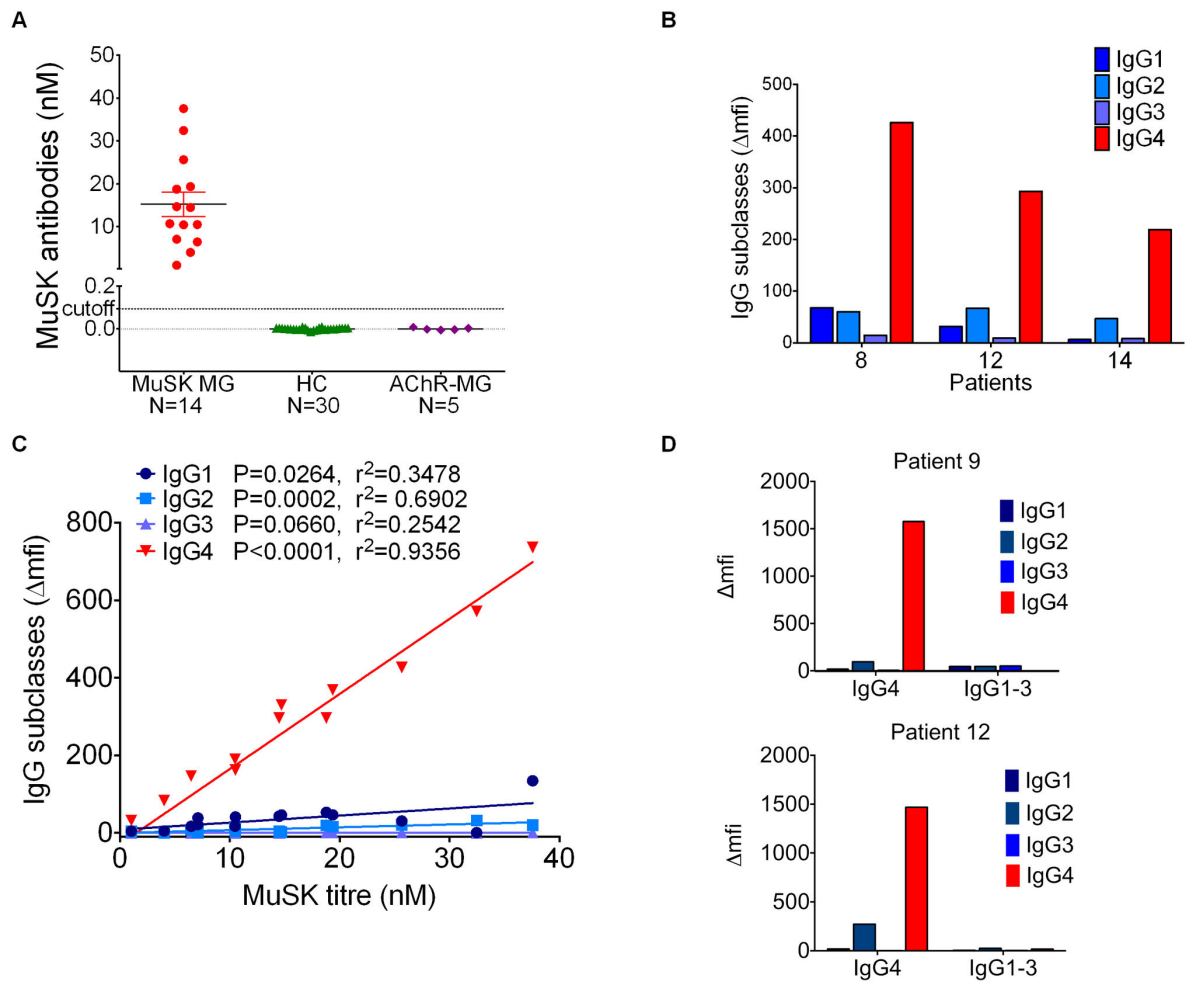
Characterisation of antibodies from MuSK-MG patients. (A) MuSK antibody titres of 14 MuSK MG patients, 30 healthy controls and 5 AChR MG patients as determined by RIA. Cut-off for MuSK antibody positivity was the mean titre of pooled healthy controls + 3x standard deviation. (B) Example of IgG subclass profiles from 3 MuSK patients. Patient plasma was incubated with HEK293 cells expressing MuSK, or to mock-transfected HEK293 cells. A subclass-specific secondary antibody was added, followed by a fluorescent tertiary antibody, and the amount of binding (mean fluorescence intensity, mfi) was measured by flow cytometry. Δmfi is the mfi obtained with cells expressing MuSK minus the mfi with mock-transfected cells. (C) Correlation between IgG subclasses, measured by flow cytometry as for (B), and MuSK antibody titres measured by RIA of 14 MuSK patient plasmas. Statistic analysis: linear regression followed by Pearson correlation (Gaussian distribution was determined by D’Agostino and Pearson test). (D) IgG1-3 and IgG4 subclasses were purified from MuSK-MG patients 9 and 12. The purity of each of these two subclass groups was analysed by flow cytometry as for (B). The IgG4 fraction for both patients contains some contamination of IgG2, but the IgG1-3 fraction is devoid of IgG4 for both patients.

The MuSK-MG patients had titres ranging from 1.03 to 37.57 nM (Figure 1A) and all were negative for AChR antibodies (data not shown). To verify that the majority of the MuSK antibodies were of the IgG4 isotype, as found previously[21], HEK293 cells expressing full length MuSK, or mock-transfected cells as a negative control, were incubated with patient antibodies before fixing and staining with subclass specific antibodies and a fluorescent tertiary antibody. The median fluorescence intensity (mfi) of the cells was measured using flow cytometry after subtraction of the binding to mock-transfected cells. All 14 patients were strongly positive for IgG4 (three representative examples are shown in Figure 1B), and the binding correlated with MuSK IgG4 antibody titre (Pearson, r^2^=0.9356, p<0.0001, Figure 1C). Although the IgG1 and 3 subclasses had much lower titres, their binding also correlated with the overall antibody titre (individual p and r^2^ values in Figure 1C). 

For further experiments, IgG4 was purified from two MuSK-MG patients from whom there was sufficient plasma, using an IgG4 affinity matrix, and the remaining IgG1-3 subclasses were purified from the flow-through. An example of elution profiles of the subclasses is shown in Figure S1A. The purity of the subclass fractions was analysed by flow cytometry (Figure 1D). Purified IgG4 contained a large quantity of MuSK-specific IgG4 and a small contamination of IgG2 antibodies. Purified IgG1-3 had only small amounts of IgG1-3, but was free of IgG4. MuSK antibody titres in each fraction were assessed by RIA (Figure S1B).

### MuSK antibodies are not endocytosed

**Figure 2:**
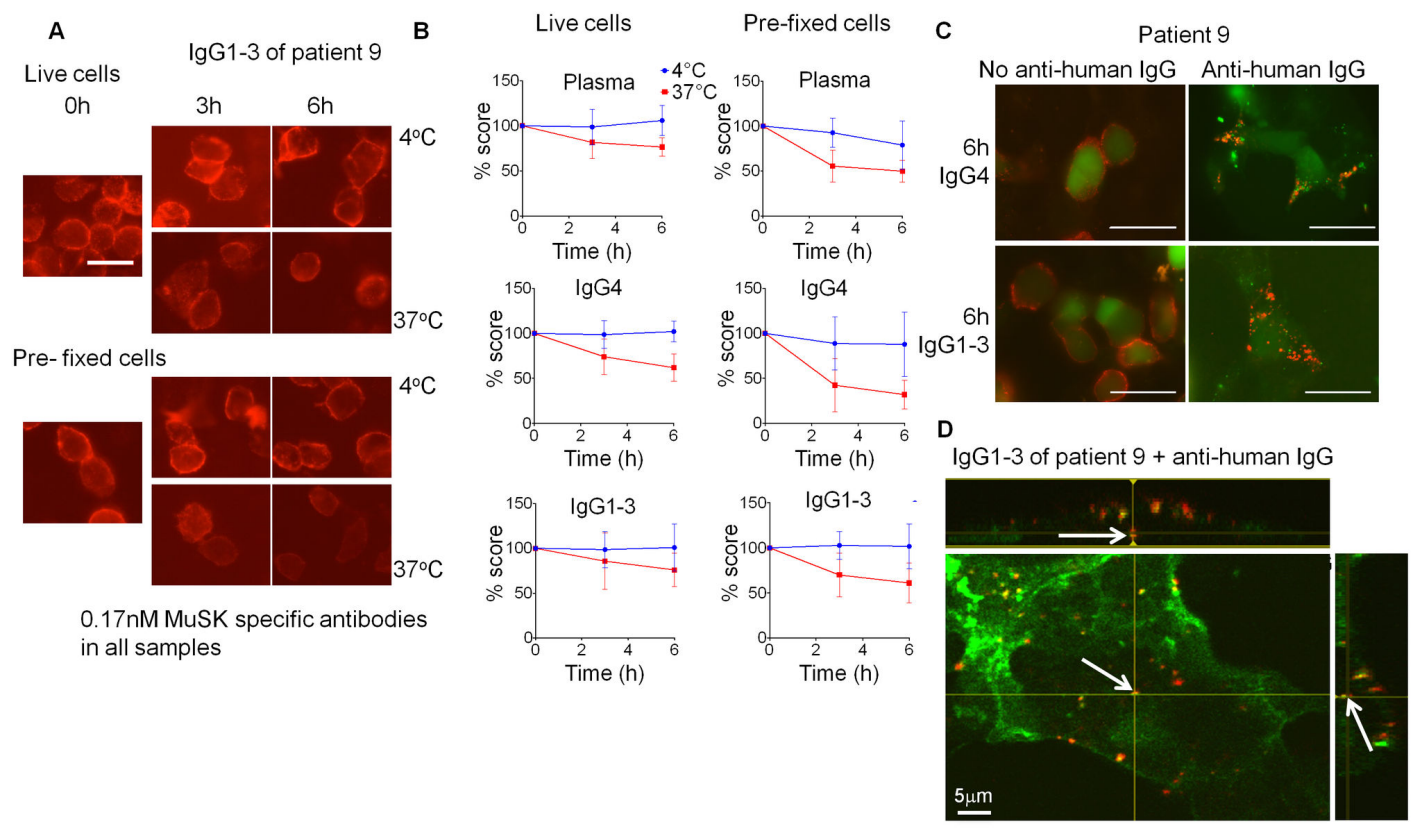
MuSK patient IgG4 or IgG1-3 do not induce endocytosis of MuSK. Patient plasma or purified IgG1-3 or IgG4 were applied at 0.17nM final concentration of MuSK antibody to HEK293 cells expressing MuSK at the cell surface. Cells were either incubated at 4°C to prevent, or at 37°C to allow, endocytosis. Patient antibody binding was visualised by addition of a secondary fluorescent anti-human antibody at the end of the experiment. (A) An example of cells that were incubated with IgG1-3 from patient 9. Robust staining was observed after 6 hours incubation at both temperatures. Scale bar=25µm. (B) Staining was scored by two individuals as described in methods, and the scores were normalised to the score at 0 hours for each condition. A slight decrease in staining was observed for cells incubated at 37°C, and pre-fixed cells also showed this reduction. (C) As a positive control for endocytosis, IgG1-3 or IgG4 was cross-linked by addition of Alexa Fluor 568-conjugated anti-human IgG prior to incubation. Example images show cells treated with patient 9 IgG4 and IgG1-3 in the presence and absence of cross-linking anti-human IgG after 6 hours incubation at 37°C. There is a clear difference when the cross-linking secondary antibody is present. Scale bar =50µm (D) Cross-linking by the secondary antibody induced internalisation of human anti-MuSK antibodies as well as MuSK-EGFP as early as 30 minutes, as observed by confocal microscopy. An example image of a cell from a confocal z-stack with orthogonal side-views is shown. The arrow shows internalised secondary Alexa Fluor 568-conjugated anti-human IgG (red) colocalised with EGFP-tagged MuSK (green). Scale bar =5µm.

To see whether binding of antibodies to MuSK led to endocytosis of the IgG-MuSK complex, HEK293 cells expressing cell-surface MuSK were incubated with patient plasma, IgG4 or IgG1-3 at 4°C and detected with anti-human IgG (binding of the purified subclass preparations, like the whole IgG, was specific because they did not bind to HEK293 cells transfected with a vector expressing only EGFP (see Figure S2)). Unbound antibodies were washed off and the cells incubated for 0-6 hours at either physiological temperature (37°C), allowing cell processes such as endocytosis to proceed, or at 4°C, which should reduce physiological processes to a minimum. At different time points, the cells were fixed and stained with fluorescence-labelled anti-human IgG. As an additional control for antibody dissociation, we used cells that were fixed prior to incubation with the MuSK IgG preparations. Plasma, IgG1-3 or IgG4 caused no significant loss of bound IgG during the first 6 hours; if anything, prefixed cells tended to lose more cell surface IgG binding than living cells perhaps because the MuSK epitope had been altered by fixation (for example see Figure 2A; all results summarised in Figure 2B). The same results were obtained after 16 hours incubation (Figure S3). Thus even the divalent IgG1-3 antibodies did not appear to be internalised. 

 To see whether the lack of endocytosis was because the antibodies were only able to bind monovalently (perhaps due to restrictions in the accessibility of the IgG1-3 epitopes), we performed the experiment at 0-6 hours in the presence of a fluorescently tagged anti-human IgG that should bind divalently to the MuSK antibodies. In this case, there was a marked change in surface IgG binding that tended to form large aggregates on the cell surface (Figure 2C). Human antibodies also appeared inside the cell as early as 10 minutes, and colocalised with MuSK-EGFP when we used cells that expressed this construct, demonstrating that both antigen and antibody were endocytosed (example shown in Figure 2D). Moreover, by this stage, MuSK and human IgG was no longer detectable at the cell surface (data not shown). Given the lack of effect on surface MuSK by patient IgG binding, and the dramatic change in the presence of divalent anti-human IgG, we concluded that the MuSK antibodies alone did not cause endocytosis of MuSK and did not pursue these experiments further.

### MuSK IgG4 and IgG1-3 reduce agrin-induced AChR clustering

C2C12 myotubes are commonly used as an *in vitro* model for AChR clustering since they contain all the post-synaptic components that are required for the formation of AChR clusters upon addition of neural agrin. Using this experimental system, antibodies from MuSK MG patients have been shown to reduce agrin-induced AChR clustering [3,23,24]. We first asked whether the effect on agrin-induced clustering is limited to the IgG4 subclass. 

**Figure 3:**
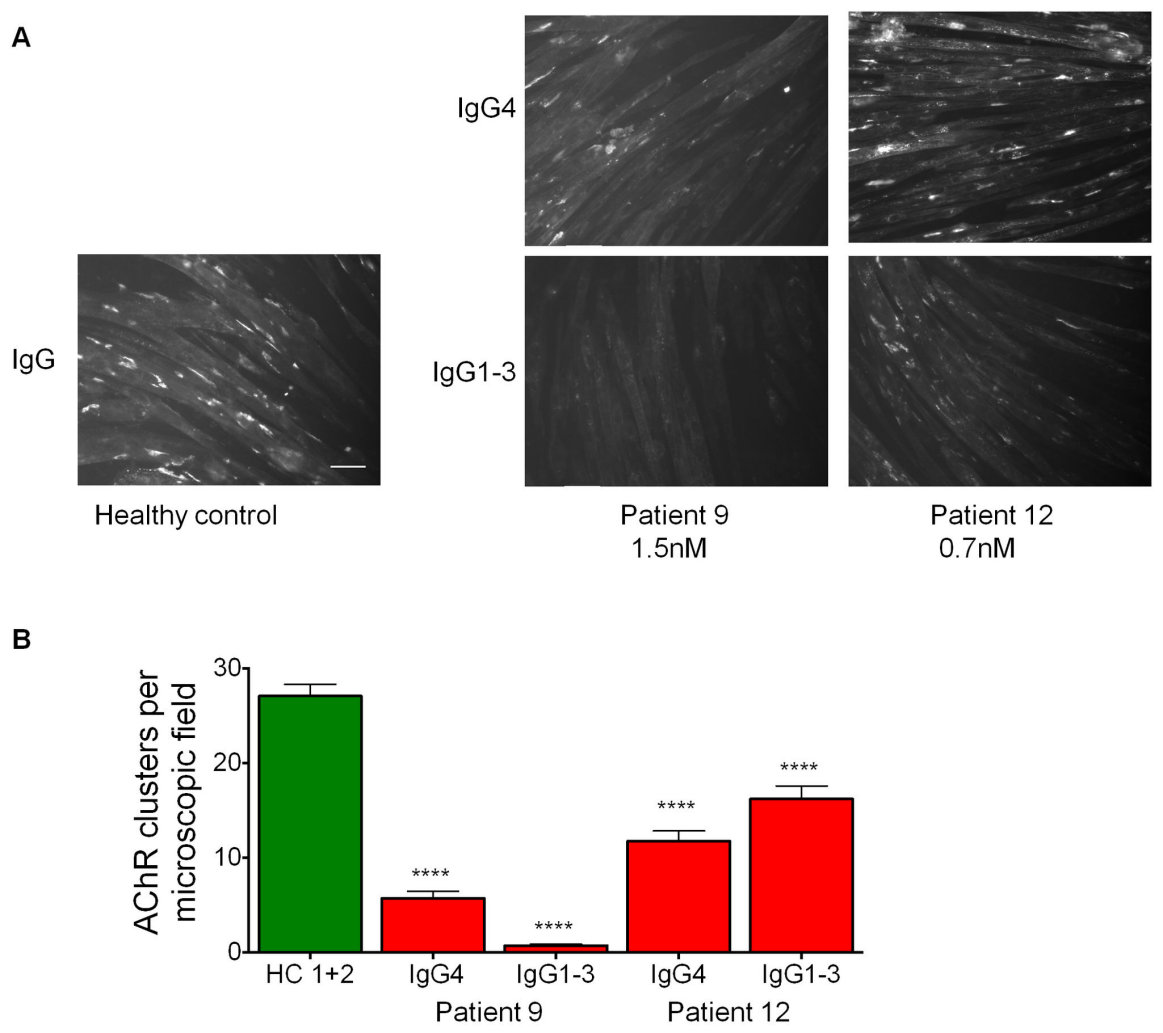
Both IgG4 and IgG1-3 subclass antibodies impair agrin-induced AChR clustering in C2C12 myotubes. C2C12 myotubes were incubated overnight with plasma from a healthy control, or with purified IgG4 or IgG1-3 at final concentrations of 0.7nM MuSK antibodies (patient 12) or 1.5nM (patient 9), in the presence of agrin. AChR clusters were stained with Alexa Fluor 594-conjugated α−bungarotoxin and 30 microscopic images at 20x magnifications were acquired. (A) Example images showing AChR clusters, scale bar =50µm. (B) Quantification of AChR clusters per field using ImageJ software (N=3). Statistical analysis: one way ANOVA (p=0.0001) followed by Bonferroni post test. **** p≤0.0001.

C2C12 myoblasts were differentiated into myotubes and incubated with IgG4 or IgG1-3 MuSK antibodies in the presence of agrin to induce the clustering of AChRs. AChRs were visualised using Alexa Fluor 594-conjugated α-bungarotoxin (examples shown in Figure 3A). The number of AChR clusters greater than 5µm in length were counted (Figure 3B). Both IgG4 and IgG1-3 significantly reduced agrin-induced AChR clustering, although the relative effects of IgG1-3 and IgG4 were different between the two patient samples. 

### MuSK antibodies interfere with the binding of LRP4 to MuSK

**Figure 4:**
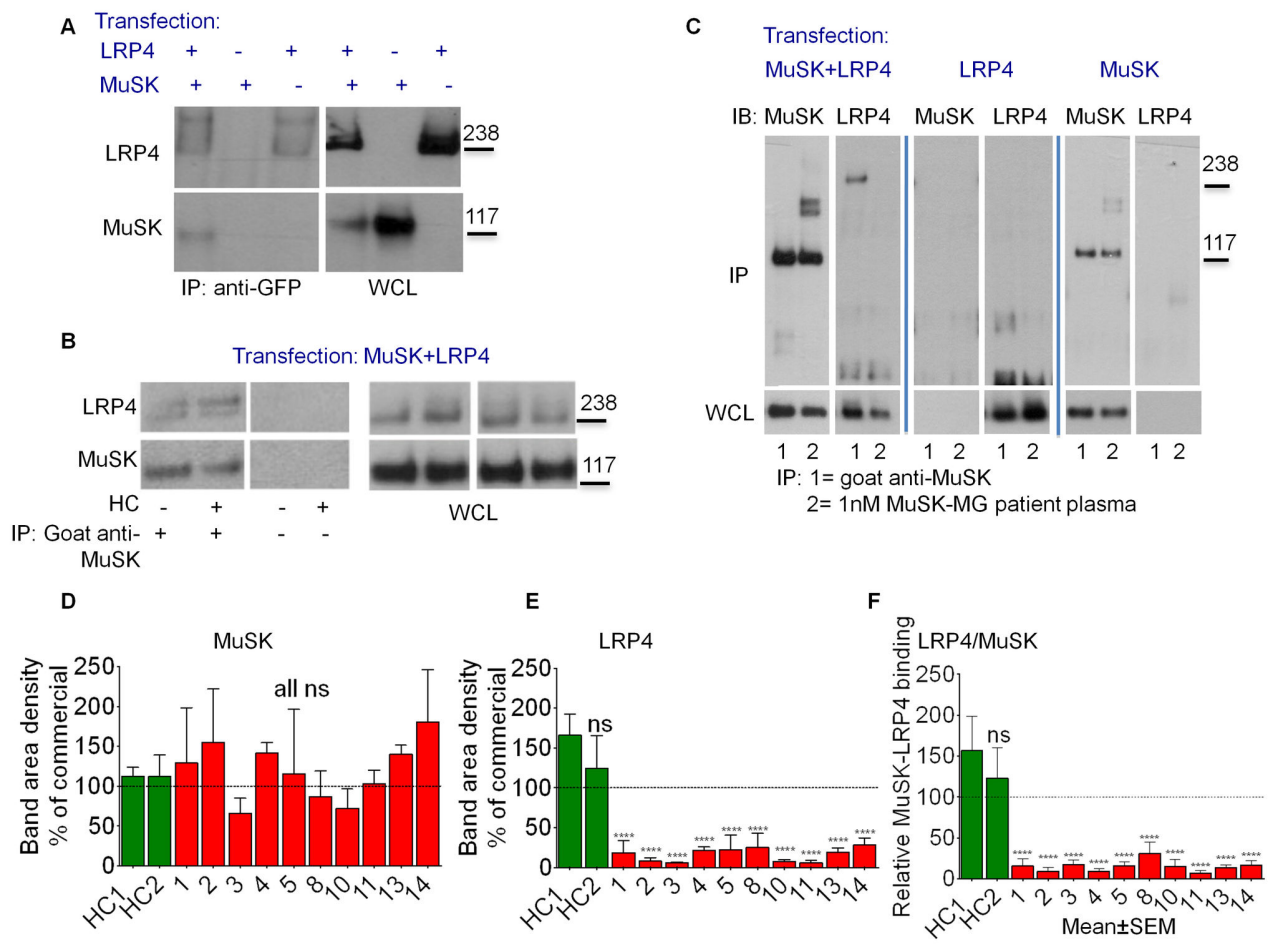
MuSK antibodies block binding between MuSK and LRP4. HEK293 cells expressing MuSK-mCherry and/or LRP4-EGFP were used to study MuSK-LRP4 binding using co-immunoprecipitation (co-IP). (A, B) Demonstration that MuSK and LRP4 interact. (A) An EGFP-specific antibody was able to co-immunoprecipitate LRP4-EGFP and MuSK-mCherry (detected with an mCherry-specific antibody) only from the surface of cells that were transfected with both LRP4-EGFP and MuSK-mCherry. (B) The MuSK-specific AF562 antibody could co-immunoprecipitate MuSK-mCherry and LRP4-EGFP only from the surface of cells transfected with both proteins. Healthy control serum (HC) did not influence this co-precipitation. (C) MuSK-MG patient plasma was able to immunoprecipitate MuSK-mCherry from the cell surface of transfected HEK293 cells, but prevented LRP4-EGFP from being co-precipitated. In the same experiments, the MuSK-specific AF562 antibody could co-precipitate LRP4-EGFP, but only in the presence of MuSK-mCherry. (D-F) The experimental set-up shown in (C) was carried out with plasma from 10 MuSK-MG patients, diluted to final MuSK antibody concentrations of 1nM (N≥3). Control immunoprecipitations were carried out with MuSK antibody AF562 in the presence of plasma from two healthy individuals (HC1 and HC2). All results were normalised to those obtained when the immunoprecipitation was carried out with only MuSK AF562 antibody (shown as a dotted line). (D) Relative area density of bands for MuSK-mCherry, (ANOVA: p=0.8273) and (E) for LRP4-EGFP (ANOVA: p<0.0001) were used to calculate the relative MuSK-LRP4 binding strength (F) (ANOVA: p<0.0001). Statistical analysis for each graph: one way ANOVA followed by Bonferroni post test, comparing each column to healthy control 1 (HC 1) column. **** p≤0.0001.

We used co-immunoprecipitation from HEK cells co-expressing MuSK and LRP4 to investigate the effect of MuSK antibodies on the interaction between LRP4 and MuSK. Full length human MuSK with a C-terminal intracellular mCherry tag (MuSK-mCherry), and full length human LRP4 with a C-terminal intracellular EGFP tag (LRP4-EGFP), were expressed at the cell surface of transfected HEK293 cells, and the live cells exposed to the antibodies before washing away unbound antibody. First we established that immunoprecipitation of LRP4 with a commercial anti-EGFP antibody could co-precipitate MuSK-mCherry (Figure 4A). Conversely, an anti-MuSK antibody (AF562) that immunoprecipitated MuSK-mCherry could co-precipitate LRP4-EGFP (Figure 4B). Since only cell-surface proteins could bind the antibodies and be immunoprecipitated, LRP4 and MuSK must be interacting on the cell surface of the transfected cells. 

When cell surface MuSK was immunoprecipitated with MuSK-MG patient plasma, however, LRP4 did not co-precipitate (Figure 4C), suggesting that the interaction between LRP4 and MuSK is blocked by patient antibodies. This was not due to some non-specific factor found in human plasma, because plasma from healthy individuals did not influence the co-precipitation of LRP4-EGFP by the commercial anti-MuSK antibody (Figure 4B). 

We carried out similar co-immunoprecipitation assays using plasma from ten MuSK-MG patients and two healthy controls. The amount of MuSK immunoprecipitated using a commercial antibody, in the absence of any human plasma, was set as 100%. The amount of patient MuSK antibody was optimised so that similar amounts of MuSK were immunoprecipitated, in order that the amount of LRP4 co-precipitated could be quantified. We also used the commercial antibody in the presence of plasma from two healthy controls to demonstrate that control plasma did not effect LRP4 co-precipitation (the concentration of the healthy plasma was the same as the highest concentration of patient plasma). Examples of western blots using plasma from patient 3 are shown in Figure S4, and from patient 4 in Figure S5. The area densities of bands corresponding to MuSK-mCherry and LRP4-EGFP on the western blots were measured, and then normalised to the density of corresponding bands immunoprecipitated with a goat anti-MuSK antibody. In all cases similar amounts of MuSK-mCherry were immunoprecipitated (Figure 4D), but all patient-treated samples reduced LRP4-EGFP co-precipitation (Figure 4E). To compensate for variations in MuSK-mCherry precipitation, relative LRP4-MuSK binding efficiency was normalised to the amount of MuSK in the immunoprecipitate (% area density of LRP4/area density of MuSK, Figure 4F). All patient IgGs substantially reduced relative LRP4-MuSK binding efficiency. 

### IgG4, but not IgG1-3, block LRP4-MuSK interaction

**Figure 5:**
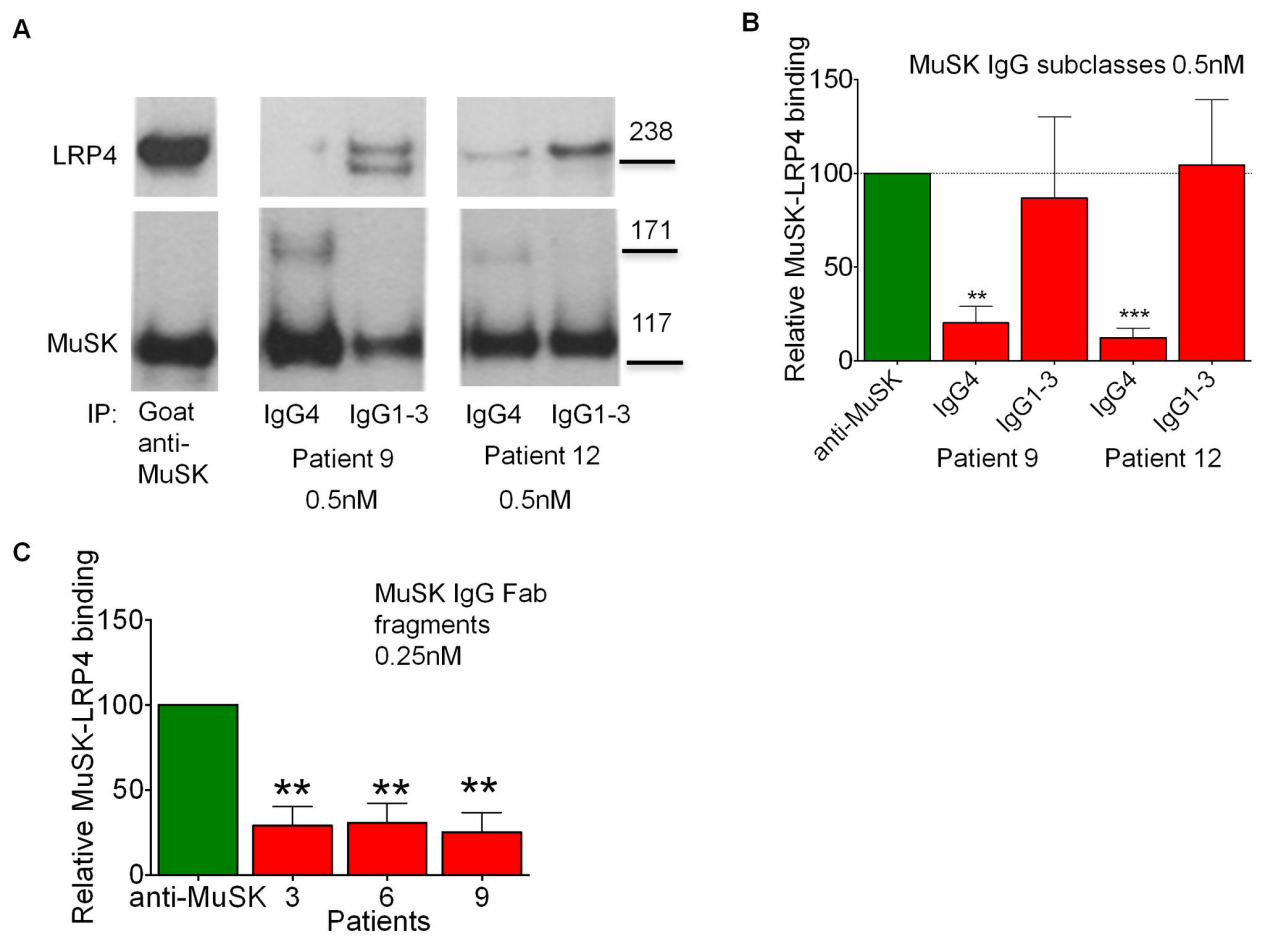
Fab fragments and IgG4, but not IgG1-3, subclass antibodies to MuSK block MuSK-LRP4 binding. (A, B) Purified IgG4 or IgG1-3 from patients 9 and 12 diluted to MuSK antibody concentration of 0.5nM, or MuSK-specific AF562 antibody, were used to immunoprecipitate MuSK-mCherry and co-precipitate LRP4-EGFP from the cell surface of transfected HEK293 cells. (A) Example Western blots probed with antibodies against MuSK-mCherry or LRP4-EGFP as indicated. (B) Quantification of relative MuSK-LRP4 binding strength, which was calculated the same way as for Figure 4F. Statistic analysis with one way ANOVA (p=0.0952) followed by Bonferroni post test, comparing data from patient samples with data using the MuSK-specific antibody for co-IP. (C) 0.25nM MuSK specific Fab fragments from patients 3, 6 and 9 were used in a similar experiment, this time using a Fab-specific secondary antibody for the co- immunoprecipitation, and the relative MuSK-LRP4 binding strength was calculated. Statistic analysis: one way ANOVA (p=0.0021) followed by Bonferroni post test, comparing each column to the commercial antibody column. ** p≤0.01, *** p≤0.001.

To investigate which MuSK subclasses were involved, we used fractionated IgG4 and IgG1-3 subclasses of patients 9 and 12 instead of whole IgG to immunoprecipitate MuSK. When equivalent levels of MuSK antibody were used, similar amounts of MuSK were precipitated by IgG4 and IgG1-3 (Figure 5A), but whereas the IgG4 preparations reduced LRP4-MuSK co-precipitation, the IgG1-3 preparations had no effect (Figure 5A,B). Purified IgG from healthy individuals did not reduce LRP4 co-precipitation (Figure S6). 

### Monovalent binding of MuSK Fab fragments reduces agrin-induced AChR clustering in C2C12 myotubes and blocks the interaction between LRP4 and MuSK

**Figure 6:**
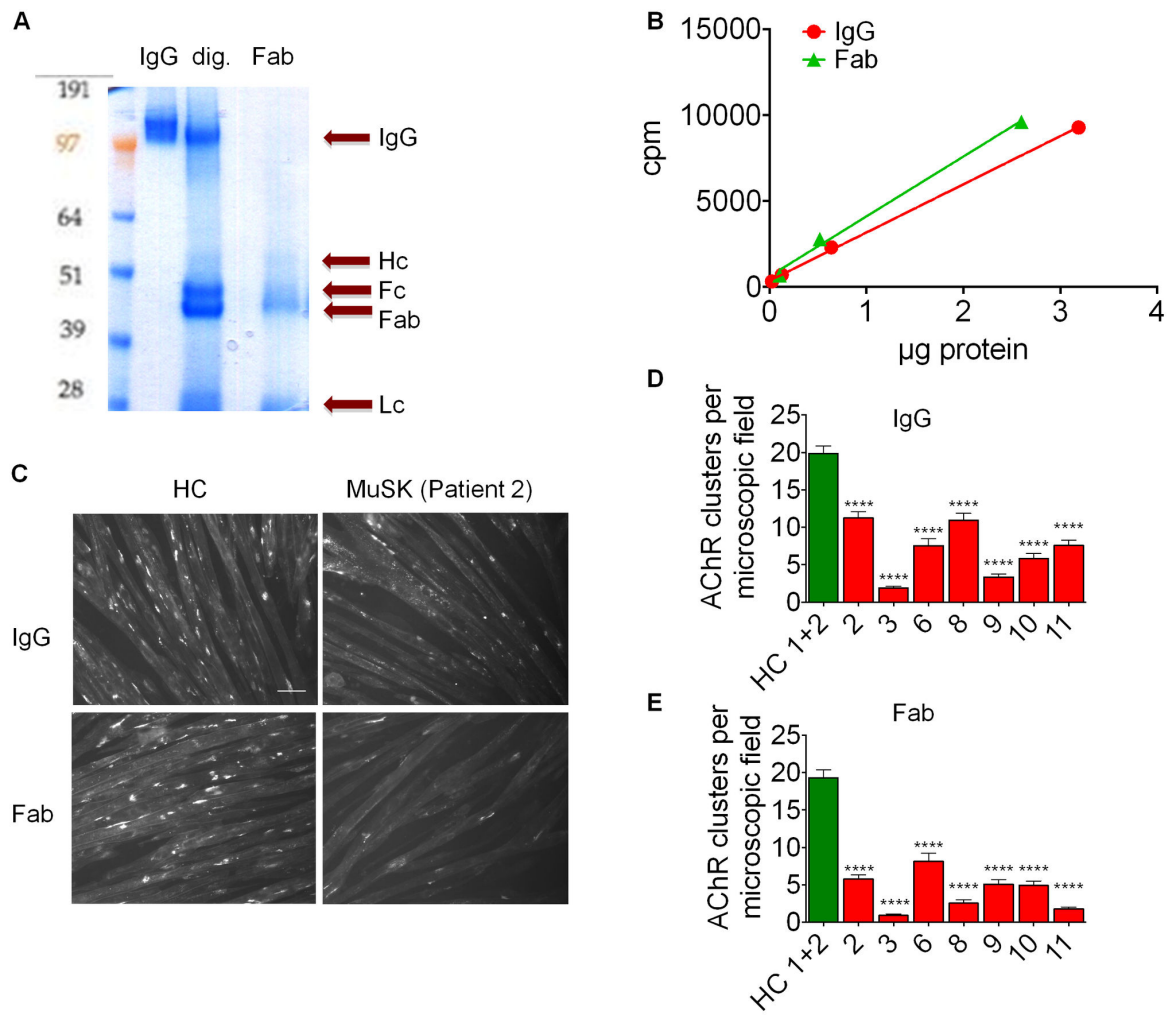
Patient derived Fab fragments reduce agrin-induced AChR clustering as well as purified IgG. Whole IgG was purified from plasma from seven MuSK-MG patients and digested to Fab fragments with papain. (A) Example of purified Fab fragments analyzed by non-denaturing gel electrophoresis and coomassie stain, showing starting material containing whole IgG, papain-digested IgG (dig.) and purified Fab fragments (Fab). Note the absence of whole IgG (IgG) in the lane containing purified Fab. Further bands represent the presence of light chain (Lc) or the Fc fragment (Fc). The absence of heavy chain, which would migrate as indicated (Hc), demonstrates complete digestion by the papain. (B) Binding of IgG and Fab fragments to radiolabelled MuSK plotted against quantity of proteins used. (C-E) The effect of Fab fragments on agrin-induced AChR clustering on C2C12 myotubes was tested. Myotubes were incubated overnight in the presence of agrin and patient or healthy control derived purified IgG or Fab fragments. For each patient, Fab fragments and IgG were used at final MuSK antibody concentrations: patient 2=0.81nM, patient 3=4.57nM, patient 6=1.37nM, patient 8=0.75nM, patient 9= 1.13nM, patient 10=0.21nM, patient 11=0.11nM. AChR clusters were visualised with Alexa Fluor 594-conjugated α-bungarotoxin, and 30 microscopic images were acquired and analyzed blinded. (C) Example images showing myotubes that were incubated with healthy control (HC) or patient 2 IgG or Fab fragments, scale bar= 50µm. (D, E) Pooled data from three experiments showing the effect of IgG or Fab fragments on AChR clustering for all patients tested. Statistical analysis: one way ANOVA followed by Bonferroni post test, each column was compared to pooled healthy control column. **** p≤0.0001 for (D) and (E).

Although we found that IgG1-3 as well as IgG4 can reduce agrin-induced clusters (Figure 3), the majority of the MuSK antibodies are IgG4 and this subclass is generally thought to be functionally monovalent, although this has not been demonstrated for MuSK specific antibodies. To see if monovalent binding is sufficient to reduce agrin-induced AChR clustering, IgG was purified from seven MuSK-MG patients or two healthy individuals and digested with papain to produce Fab fragments which were then examined for absence of whole IgG by gel electrophoresis and coomassie staining (Figure 6A). Functional binding of MuSK was assessed by RIA (Figure 6B shows example data from one patient). Fab fragments were then analysed for their effects on agrin-induced AChR clustering (Figure 6C). Data from myotubes treated with Fab fragments from two different healthy individuals were pooled and compared to the patient samples, and all samples were anonymised before application to the myotubes. Whole IgG and Fab fragments from all seven MuSK-MG patients substantially reduced AChR clustering (Figure 6D,E), indicating that monovalent binding of antibodies is sufficient to impair AChR clustering (as shown previously for experimentally-induced Fab fragments [25]) . 

 Similarly, we tested whether the Fabs inhibited LRP4-MuSK binding, using co-immunoprecipitation with Fab fragments and Fab specific anti-human IgG rather than anti-IgG, so that only those MuSK molecules that were bound to Fab fragments were immunoprecipitated. Fab fragments from three different MuSK-MG patients efficiently immunoprecipitated MuSK-mCherry, but did not co-precipitate LRP4 compared to the commercial MuSK antibody (Figure 5C). Fab fragments from healthy controls did not prevent co-precipitation of LRP4 (Figure S6).

### MuSK Ab positive plasma disperses agrin-independent Dok7-induced AChR clusters

**Figure 7:**
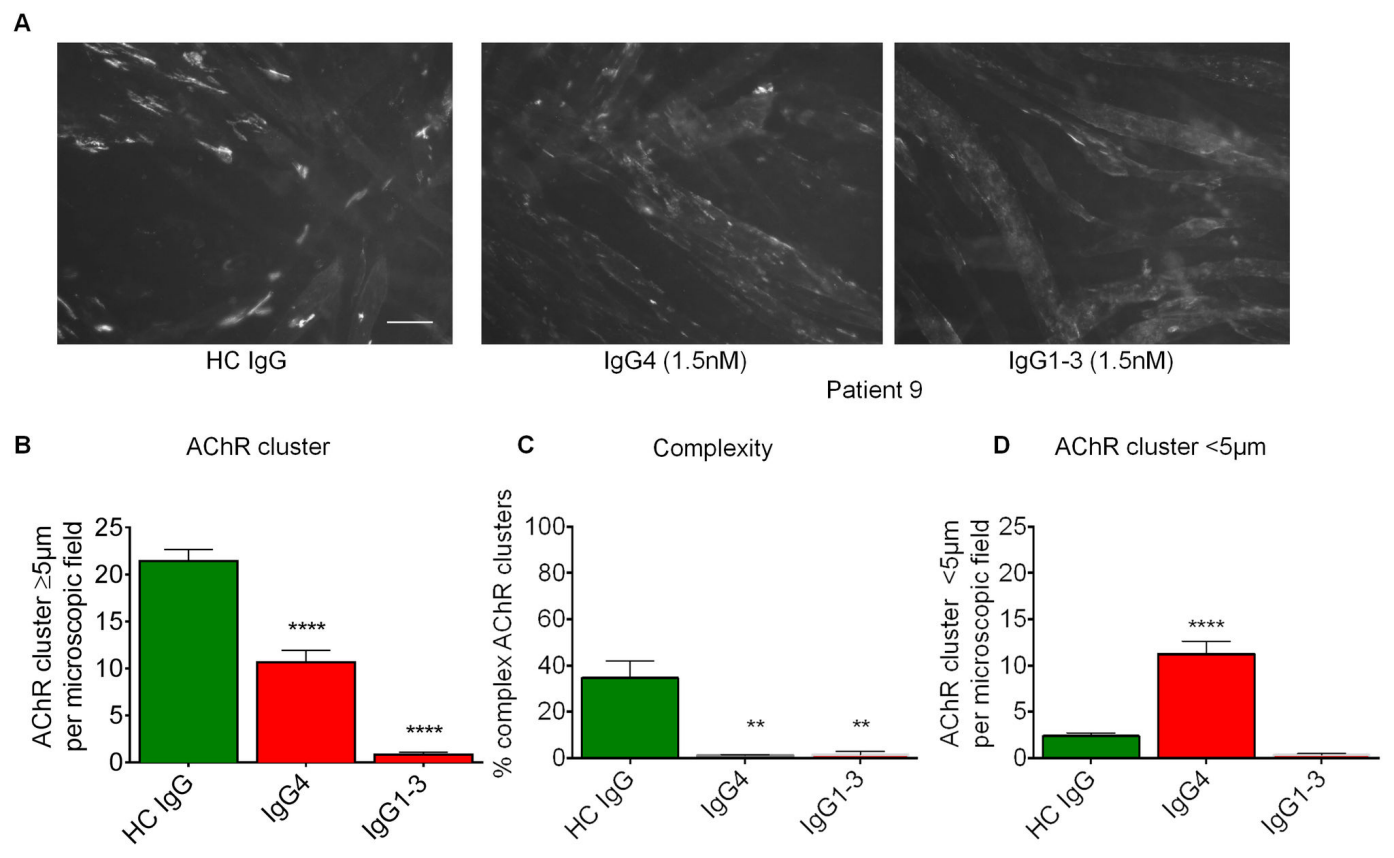
MuSK MG patient IgG1-3 and IgG4 disrupt Dok7-induced AChR clustering on C2C12 myotubes. Myotubes were incubated overnight with 1.5nM MuSK specific IgG1-3 or IgG4 from patient 9. (A) Representative images of AChR clusters, visualised with Alexa Fluor 594-conjugated α-bungarotoxin. (B) Number of AChR clusters ≥5µm was quantitated, average of three experiments. (C) The proportion of complex AChR clusters (perforated, c-shaped and branched - see Figure S7) was calculated. (D) Number of AChR clusters <5µm was measured. One way ANOVA (p<0.0001) followed by Bonferroni post test. ** p≤0.01, **** p≤0.0001.

Finally, we asked whether MuSK antibodies act only by inhibiting LRP4-MuSK interaction, or by altering MuSK function directly. If Dok7 is over-expressed in C2C12 myotubes, AChR clustering occurs in the absence of agrin [8,26]. In this situation the requirement for agrin binding to LRP4 is by-passed, because over-expressed Dok7 induces dimerisation and activation of MuSK independent of agrin-LRP4-MuSK. We therefore used the stable C2C12 myoblast cells overexpressing human Dok7 [26], differentiated them into myotubes and then applied 1.5nM purified MuSK IgG1-3 or IgG4 from patient 9. This level of IgG1-3 was similar to that in the original plasma. As a control we included purified IgG from healthy control 2 at a similar IgG concentration to that of patient 9. Example images are shown in Figure 7A. 

Myotubes treated with healthy control IgG had approximately 20 AChR clusters of ≥5µm per field. By contrast, very few clusters of this size were observed on myotubes treated with IgG1-3 purified from MuSK-MG patient 9 (Figure 7B), while the IgG4 preparation reduced the number of clusters but only to approximately 10 per field. Up to 35% of the AChR clusters on myotubes treated with healthy control IgG were of a complex shape, perforated, c-shaped or branched (see 8,27; examples shown in Figure S7), whereas both IgG1-3 and IgG4 from patient 9 reduced the proportion of complex AChR clusters to less than 1% (Figure 7C). IgG4 also appeared to break-up the AChR clusters into visible clusters of less than 5µm in length (Figure 7D); this was not seen with IgG1-3 MuSK antibodies suggesting even greater dispersal of the AChRs. When we subsequently performed the experiment on newly-infected C2C12 cells with our limited amounts of purified subclasses from patient 12 (n=2; Figure S8) both subclass preparations dispersed the AChR clusters effectively.

## Discussion

Over a decade has passed since the discovery of MuSK antibodies in myasthenia [3] but, although *in vivo* transfer and active immunization studies have confirmed the pathogenicity of MuSK antibodies, whether MuSK antibodies reduce MuSK function, reduce MuSK levels, or damage the structure and function of the neuromuscular junction by other mechanisms is still unclear. MuSK antibodies are mainly IgG4, an antibody subclass that is mainly monovalent and which would not, therefore, be expected to induce endocytosis of the target antigen, excluding one mechanism by which antibodies to membrane antigens cause loss of function. IgG1-3 MuSK antibodies, by contrast, are divalent but less prevalent and have been considered to be non-contributory in MuSK-MG. Although we could not find evidence for MuSK endocytosis, we show that the MuSK IgG antibodies block the essential interaction between LRP4 and MuSK. Moreover, the latter did not require divalent binding since both purified IgG4 and monovalent IgG Fab fragments also inhibited LRP4 binding to MuSK. MuSK IgG1-3 antibodies, however, did not inhibit the LRP4-MuSK interaction, but they too reduced AChR clustering in C2C12 myotubes. Moreover, both IgG4 and IgG1-3 MuSK antibodies dispersed preformed clusters that form in Dok7 over-expressing C2C12 myotubes. These observations suggest two, possibly independent, mechanisms by which MuSK antibodies lead to dispersion and loss of AChRs at the neuromuscular junction, namely prevention of LRP4 binding to MuSK and direct inhibition of agrin-independent MuSK activity. 

IgG4 is the predominant subclass of the antibodies that recognises MuSK [21] and disease severity correlated with IgG4 levels in individual patients [28], as well as with total IgG MuSK antibody [29]. A detailed study of electrophysiological changes in MuSK IgG-injected (immune deficient) mice implicated IgG4, but not IgG1-3, as the pathogenic entity [19]. IgG4 is an unusual subclass and contributes only 5% of total IgG [30]. The IgG4 Fc domain does not bind C1q and hence does not activate complement, and IgG4 binds poorly to Fc-γ receptors I, II and III [31], limiting its ability to stimulate phagocytic or cytotoxic reactions. Intriguingly, IgG4 can split in half and recombine with other half molecules to form chimeric antibodies with two different antigen specificities [32], resulting in antibodies that would be effectively monovalent and not capable of endocytosing their target antigen by the usual mechanisms of divalent cross-linking [33,34]. Another curiosity is that IgG4 antigen-specific antibodies usually arise only after long term exposure to antigens, with affinity maturation and subclass switching, and are likely to be protective, as in beekeepers [35,36]. Whether IgG4 MuSK antibodies are all monovalent and why these are the main antibody subclass in MuSK-MG, which is an acquired and often relatively acute-onset disease, is unexplained. 

Nevertheless, the characteristics of IgG4 suggest that the effects of MuSK antibodies will not involve endocytosis, complement deposition or cellular cytotoxicity and are more likely to involve direct effects on function. We found that antibodies from MuSK-MG patients can disrupt the LRP4-MuSK interaction *in vitro*. LRP4 is the receptor for agrin [37,38], and stimulates binding of LRP4 to the first Ig domain of MuSK [39], an interaction that is crucial for the formation and maintenance of AChR clustering at the neuromuscular junction. A previous study [40] did not find inhibition of LRP4 binding to MuSK but used a solid-phase assay rather than the more physiological assay, involving cell surface expression of the two proteins in HEK cells, that we used here. In our hands, purified IgG, Fab fragments and purified IgG4 all inhibited the LRP4-MuSK interaction and also inhibited agrin-induced AChR clustering on myotubes, which is consistent with previous findings that Fab fragments from an immunized rabbit model were sufficient to reduce AChR clustering [25]. Taken together these findings imply that neither divalent antibodies nor the Fc domain of MuSK-IgG are required for this pathogenic mechanism. The loss of LRP4-MuSK interaction may either stem directly from a block of the LRP4 binding site or, indirectly, by influencing the MuSK conformation that allows LRP4 binding to MuSK. 

IgG1-3 MuSK antibodies, by contrast, are present at relatively low levels in MuSK-MG patients, and have been considered non-pathogenic [19]. However, while IgG1-3 antibodies did not inhibit binding of LRP4 to MuSK, we found efficient reduction of agrin-induced AChR clustering on C2C12 myotubes by IgG1-3 as well as the IgG4 preparations. The relative effects of the IgG4 and 1-3 preparations from patients 9 and 12 differed but this might reflect heterogeneity of the antigenic epitopes between the patients, as is common, for instance, in AChR antibody myasthenia [41]. Moreover, they were at somewhat different stages of the disease course, as Patient 9 was at the peak of their severity, whereas patient 12 was already greatly improved following treatments; treatments could influence the efficacy of different antibodies. 

During AChR clustering, LRP4 binds to MuSK and stimulates a change in MuSK conformation (particularly in, but not necessarily restricted to, the MuSK Ig-like first domain which is a major target for MuSK antibodies [21]), thus allowing MuSK dimerisation and autophosphorylation of Y553 [42,43]. A possible explanation for the effects of the IgG1-3 antibodies on agrin-induced clustering was that they act directly to impair these functions of MuSK. To address this we used Dok7 overexpressing C2C12 myotubes. In these cells, MuSK activation is stimulated directly by Dok7 in the absence of agrin, presumably bypassing the need for LRP4-MuSK interaction [8,26]. AChR clusters are already formed on the myotubes prior to application of the antibodies, thus representing conditions very similar to the adult neuromuscular junction in patients (in contrast to application of antibodies before or concurrent with agrin which is more a model of changes that could occur during development). IgG1-3 caused complete dispersal of preformed AChR clusters, providing evidence that these antibodies can affect MuSK function independent of agrin binding to LRP4. Alternatively MuSK IgG1-3 might interfere with the interaction between MuSK and one or more of its other binding partners. COLQ binding to MuSK is thought to be important in NMJ development *in vivo* [44], although its influence on AChR clustering in cultured myotubes has not been reported. Interestingly, IgG4 MuSK antibodies also caused some dispersal of AChR clusters. This was unexpected since Dok7-induced AChR cluster formation should not be dependent on the interaction between LRP4 and MuSK, and suggests that the IgG4 antibodies also directly affect MuSK activity, perhaps by preventing a conformational change in MuSK that is required for downstream activation as well as for LRP4 binding. 

Our data provide evidence that antibodies in the IgG1-3 fraction also contribute to the pathogenesis of the disease, even though they are present in patient sera at lower levels than IgG4. This is in contrast to others’ work where passive transfer of IgG4 but not IgG1-3 into mice induced physiological evidence of MG [19]. However, MuSK-specific IgG1-3 antibodies in that study were undetectable in the mouse sera, which is not representative of the situation in MuSK-MG patients where these subclasses can be easily measured, as we show here and previously [49]. IgG1-3 subclasses are divalent and should be capable of inducing endocytosis of MuSK. However, we could not detect endocytosis in transfected HEK293 cells at the IgG concentration applied, and it may be that the binding sites for the IgG1-3 antibodies do not allow inter-MuSK cross-linking, which occurred only when we added a divalent anti-human IgG. These results contrast with those published by Cole et al (2010) who described endocytosis in transfected C2C12 myoblasts [20]. A possible explanation is that patient antibody-mediated endocytosis of MuSK requires the environment of muscle cells, although this seems unlikely since many antibodies internalise their antigen from the surface of HEK cells (eg antibodies to AChR, AQP4, glycine receptors (A Vincent, P. Waters, A. Carvajal, unpublished results)). Alternatively, antibodies from only a subset of MuSK-MG patients might be capable of inducing endocytosis. Although MuSK antibodies have been suggested to react predominantly within the Ig-like domains [21], a recent publication suggests the presence of antibodies against the CRD (juxta membrane) domain in a patient subgroup [45], which could be important for AChR clustering [46] and further MuSK endocytosis [47]. These antibodies may produce loss of MuSK in C2C12 cells in a manner that differs from the process that occurs when antibodies cross-link their antigens on the surface of other cells. Injection of mice with IgG1-3 at concentrations similar to those seen in patients could well induce pathological defects and should be performed in future work.

One unifying hypothesis is that the MuSK antibodies are initially relatively low affinity IgG1-3 subclasses but that these do not compete effectively with LRP4 to achieve inhibition of MuSK function and the patients may remain clinically unaffected. Only as the antibodies mature in affinity, in parallel with a switch of the majority to the IgG4 subclass, do they compete adequately with LRP4 and begin to produce clinically-relevant changes. It is possible that the monovalency of the IgG4 antibodies may have an advantage when it comes to inhibition of LRP4 binding. It would be interesting to see detailed epitope mapping and structural studies to address this possibility and to assess whether the IgG1-3 antibodies are lower affinity and have the same or different epitope specificity.

 IgG1-3 MuSK antibodies should be capable of activating complement independent of the effects described here. Complement has not so far been detected in mouse models of MuSK-MG, but this may be because mouse complement is not well activated by human IgG [48]. In patients there is some evidence that complement may play a role. Sera from MuSK-MG patients were able to activate complement on MuSK-transfected HEK cells *in vitro* [49], and complement breakdown products were elevated in MuSK-MG patients’ sera [50]. There is considerable variation in MuSK mRNA expression in different adult skeletal muscles [51] which may explain different susceptibility to muscle weakness in facial muscles compared to limb muscles. It could be that at some endplates, perhaps those in highly vulnerable bulbar and facial muscles, the density of MuSK is high enough for the IgG1-3 antibodies to cause complement-mediated damage, in addition to the direct inhibitory effects of both IgG4 and IgG1-3 MuSK antibodies. The possibility that IgG1-3 subclass antibodies can be pathogenic could be clinically relevant. Rituximab, which is emerging as a useful treatment in MuSK myasthenia gravis [52], acts predominantly on IgG4 specific B cells in IgG4 related systemic diseases [53,54]. Furthermore, Rituximab stimulates CD20+ B cells to increase production of type I interferons [55], which have been implicated in class switching in autoreactive B cells (reviewed by [56]). Although clinical studies for rituximab use in MuSK myasthenia gravis are promising [57] it should be kept in mind that the potentially pathogenic IgG1-3 subclass antibodies may not be targeted. 

## Supporting Information

Figure S1 **Example of IgG4 and IgG1-3 purification.** (A) Example elution profiles of IgG4 and IgG1-3. (B) Titres and protein concentrations of the antibody subclass fractions for two MuSK-MG patients and IgG from two healthy individuals.
(TIF)

Figure S2 **IgG4 and IgG1-3 from MuSK-MG patients specifically recognise MuSK in a cell-based assay.** HEK29 cells were transfected with a construct expressing MuSK and EGFP (pG-MuSK) or EGFP only (pG-). Cells were incubated with purified antibodies from patient 9 and detected using a secondary fluorescent anti-human antibody (red). (A) IgG4 and (B) IgG1-3 only recognised cells expressing MuSK with EGFP and not cells expressing EGFP alone.
(TIF)

Figure S3 **Prolonged exposure to MuSK patient IgG4 or IgG1-3 does not induce loss of bound IgG.** Patient plasma or purified IgG1-3 or IgG4 were applied to HEK293 cells expressing MuSK at the cell surface. Cells were either incubated at 4°C to prevent, or at 37°C to allow, endocytosis. (A) Staining of cells with a secondary fluorescent anti-human antibody (red) showed a decrease in patient antibody binding after 16 hours at 37°C. However, this was not due to endocytosis because pre-fixed cells also showed a similar reduction. (B) Level of fluorescence was scored and normalised to time point 0 hours.
(TIF)

Figure S4 **Example of a western blot from an immunoprecipitation experiment using HEK293 cells expressing both MuSK-mCherry and LRP4-EGFP.** (A) The commercial anti-MuSK antibody and patient 3 plasma both immunoprecipitated MuSK but only the commercial anti-MuSK antibody was able to co-immunoprecipitate LRP4. (B) Western blot analysis of whole cell lysates demonstrated that similar amounts of MuSK and LRP4 were expressed and therefore input into the immunoprecipitation experiment. An anti-mCherry antibody was used to detect MuSK-mCherry and an antibody against EGFP was used to detect LRP4-EGFP in the immunoblot (IB) as indicated.
(TIF)

Figure S5 **Scans of whole western blots used to generate Figure 3C.** (A, B) Western blots using an anti-mCherry antibody to detect MuSK-mCherry or (C, D) an antibody against EGFP to detect LRP4-EGFP. (A, C) Western blots of the immunoprecipitations and (B, D) of the whole cell lysates. Cells were transfected with MuSK-mCherry and/or LRP4-EGFP as indicated. Antibodies used in IP: 4= patient 4 plasma, M= goat anti-MuSK antibody.
(TIF)

Figure S6 **Healthy control IgG and healthy control Fab fragments do not interfere with MuSK-LRP4 binding.** MuSK-mCherry was immuno-precipitated from the cell surface of HEK293 cells transfected with MuSK-mCherry and LRP4-EGFP using a goat anti-MuSK antibody alone, or in the presence of healthy control IgG (1,2) or healthy control Fab fragments (4,5) at volumes equal to those of patient samples used in experiments. (A) Western blot using an anti-mCherry antibody to detect MuSK-mCherry, and (B) Western blot with anti-GFP to detect LRP4-EGFP. Similar amounts of MuSK-mCherry and LRP4-EGFP were precipitated in all samples.
(TIF)

Figure S7 **Examples of complex AChR clusters that were induced by overexpressing Dok7 in C2C12 myotubes and stained using Alexa Fluor 594-conjugated α-bungarotoxin.** (A) Arrows indicate perforated clusters. (B) c-shaped clusters. (C) Branched clusters.
(TIF)

Figure S8 **MuSK-MG patient IgG1-3 and IgG4 disrupt Dok7-induced AChR clustering on C2C12 myotubes.** Myotubes were incubated overnight with 0.11nM MuSK specific IgG1-3 or IgG4 from patient 12. Results are the average of two experiments. (A) Number of AChR clusters ≥5μm was quantitated. (B) The proportion of complex AChR clusters (perforated, c-shaped and branched - see Figure S7) was calculated. (C) Number of AChR clusters <5µm was measured. One way ANOVA (p<0.0001) followed by Bonferroni post test. * p≤0.05, ** p≤0.01, **** p≤0.0001.
(TIF)
