# Supplementary figures and images for: MuSK Myasthenia Gravis IgG4 Disrupts the Interaction of LRP4 with MuSK but Both IgG4 and IgG1-3 Can Disperse Preformed Agrin-Independent AChR Clusters
Source: PLoS One. 2013 Nov 7;8(11):e80695. doi: 10.1371/journal.pone.0080695 (PMC3820634; doi:10.1371/journal.pone.0080695)

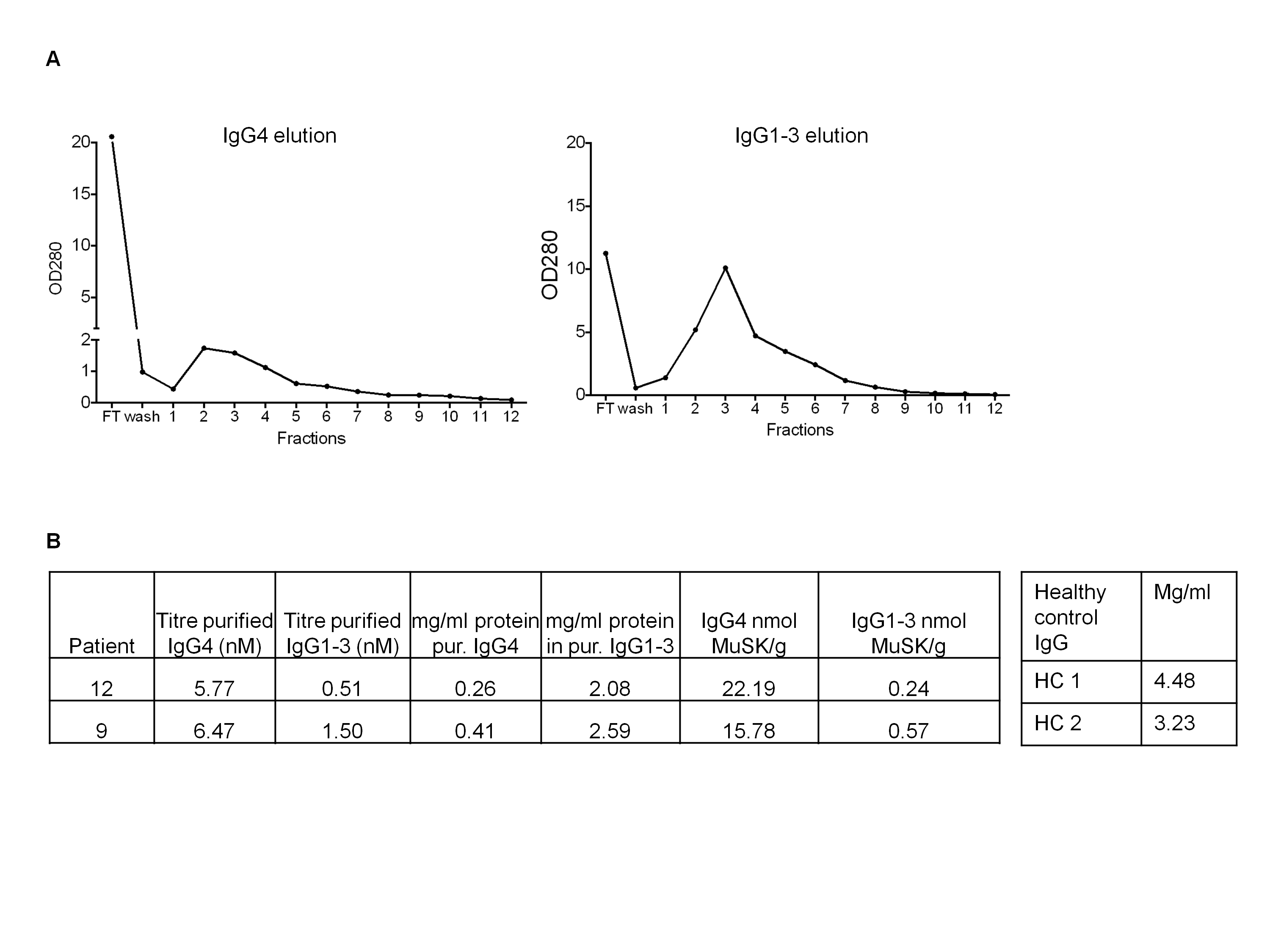

Supplement: Figure S1 — Example of IgG4 and IgG1-3 purification. (A) Example elution profiles of IgG4 and IgG1-3. (B) Titres and protein concentrations of the antibody subclass fractions for two MuSK-MG patients and IgG from two healthy individuals. (TIF) [file pone.0080695.s001.tif]

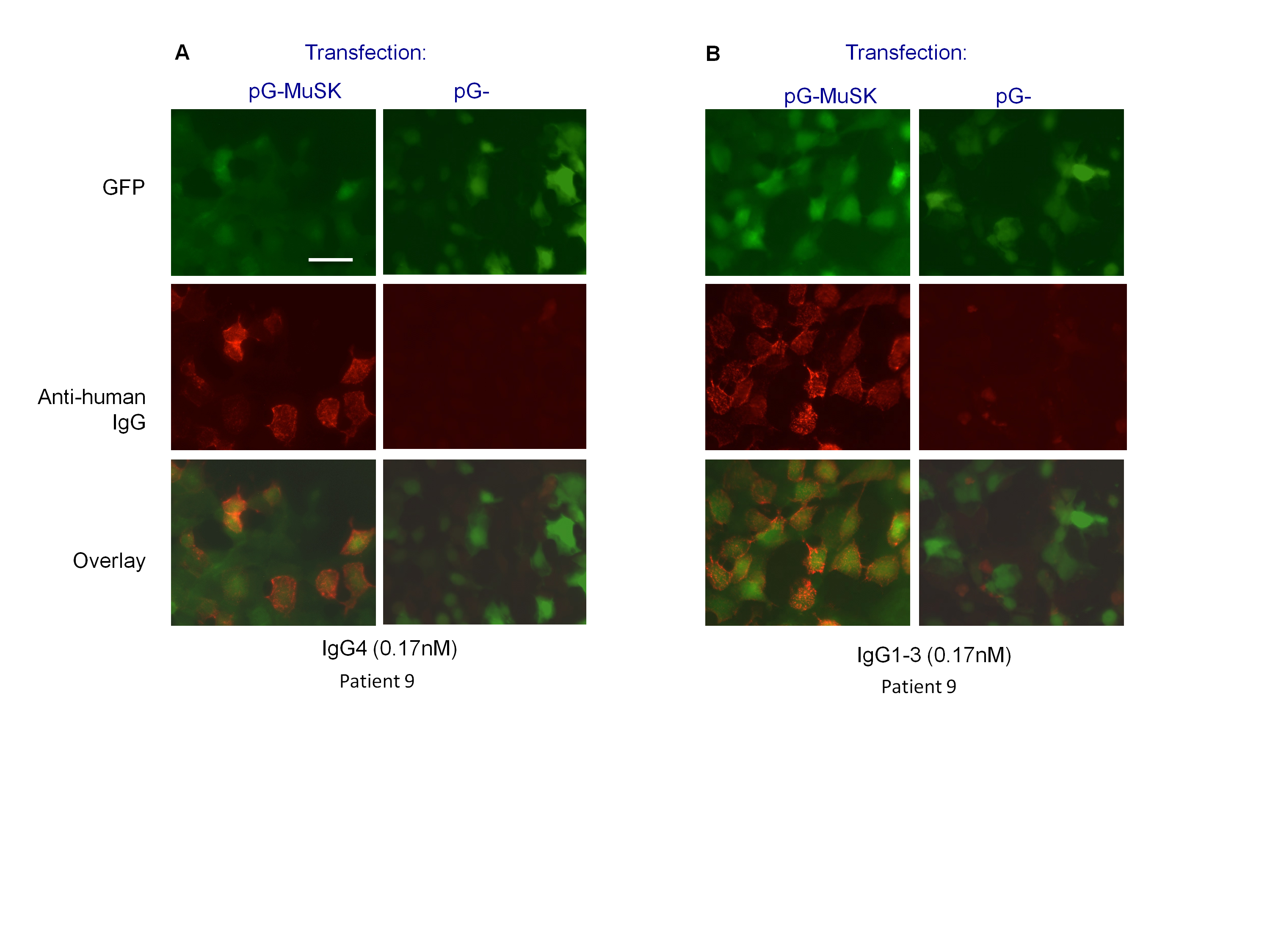

Supplement: Figure S2 — IgG4 and IgG1-3 from MuSK-MG patients specifically recognise MuSK in a cell-based assay. HEK29 cells were transfected with a construct expressing MuSK and EGFP (pG-MuSK) or EGFP only (pG-). Cells were incubated with purified antibodies from patient 9 and detected using a secondary fluorescent anti-human antibody (red). (A) IgG4 and (B) IgG1-3 only recognised cells expressing MuSK with EGFP and not cells expressing EGFP alone. (TIF) [file pone.0080695.s002.tif]

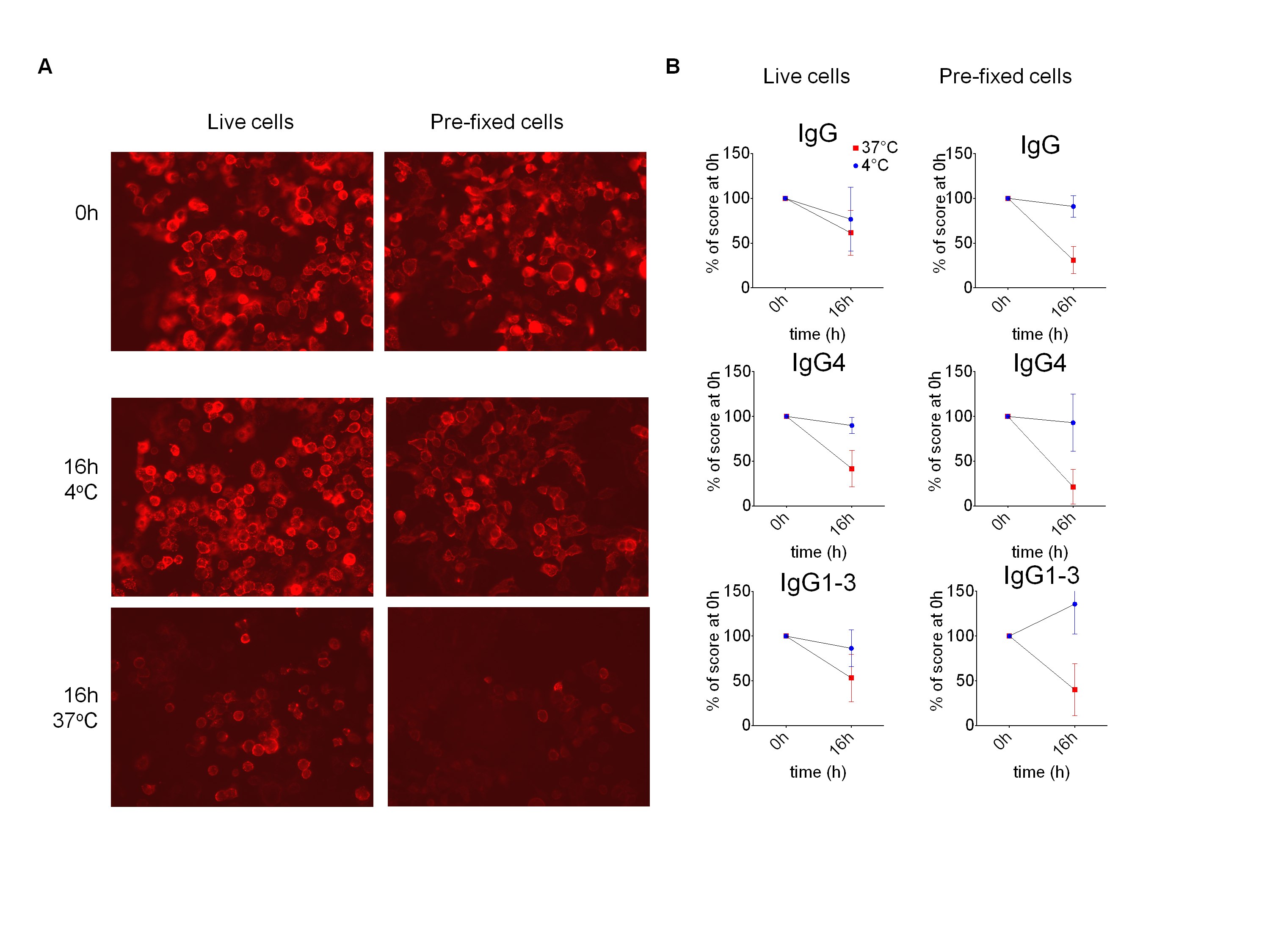

Supplement: Figure S3 — Prolonged exposure to MuSK patient IgG4 or IgG1-3 does not induce loss of bound IgG. Patient plasma or purified IgG1-3 or IgG4 were applied to HEK293 cells expressing MuSK at the cell surface. Cells were either incubated at 4°C to prevent, or at 37°C to allow, endocytosis. (A) Staining of cells with a secondary fluorescent anti-human antibody (red) showed a decrease in patient antibody binding after 16 hours at 37°C. However, this was not due to endocytosis because pre-fixed cells also showed a similar reduction. (B) Level of fluorescence was scored and normalised to time point 0 hours. (TIF) [file pone.0080695.s003.tif]

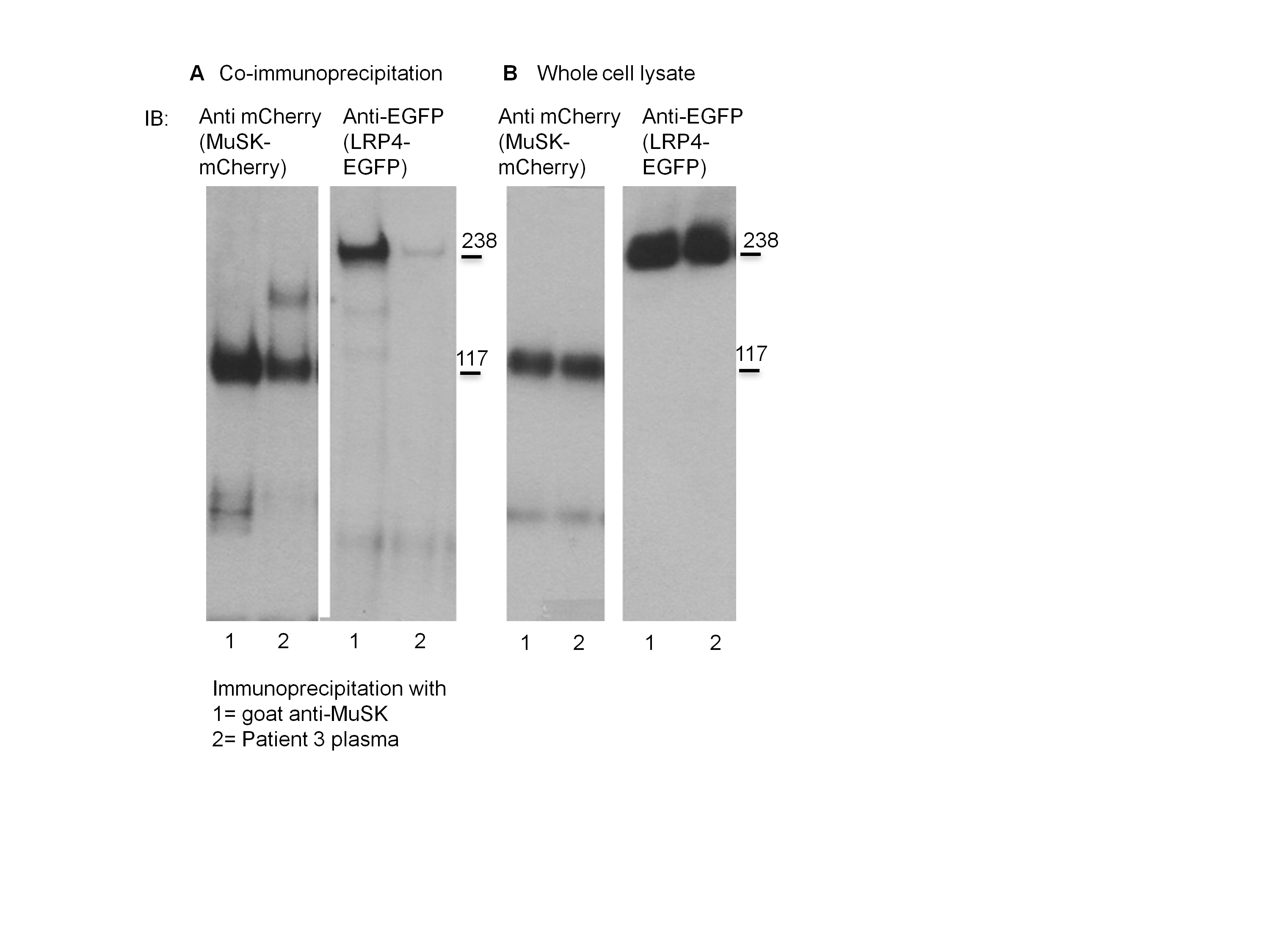

Supplement: Figure S4 — Example of a western blot from an immunoprecipitation experiment using HEK293 cells expressing both MuSK-mCherry and LRP4-EGFP. (A) The commercial anti-MuSK antibody and patient 3 plasma both immunoprecipitated MuSK but only the commercial anti-MuSK antibody was able to co-immunoprecipitate LRP4. (B) Western blot analysis of whole cell lysates demonstrated that similar amounts of MuSK and LRP4 were expressed and therefore input into the immunoprecipitation experiment. An anti-mCherry antibody was used to detect MuSK-mCherry and an antibody against EGFP was used to detect LRP4-EGFP in the immunoblot (IB) as indicated. (TIF) [file pone.0080695.s004.tif]

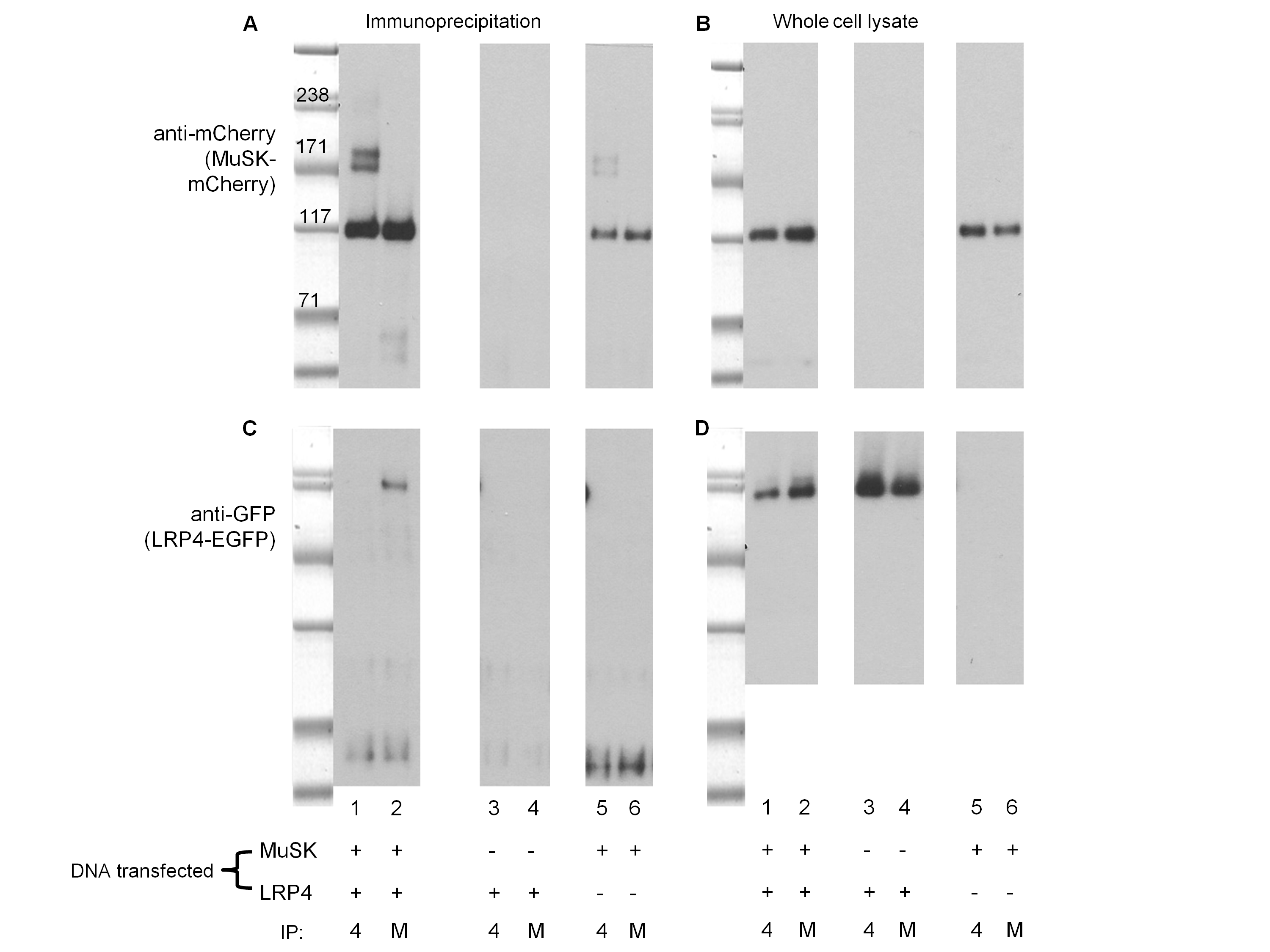

Supplement: Figure S5 — Scans of whole western blots used to generate Figure 3C. (A, B) Western blots using an anti-mCherry antibody to detect MuSK-mCherry or (C, D) an antibody against EGFP to detect LRP4-EGFP. (A, C) Western blots of the immunoprecipitations and (B, D) of the whole cell lysates. Cells were transfected with MuSK-mCherry and/or LRP4-EGFP as indicated. Antibodies used in IP: 4= patient 4 plasma, M= goat anti-MuSK antibody. (TIF) [file pone.0080695.s005.tif]

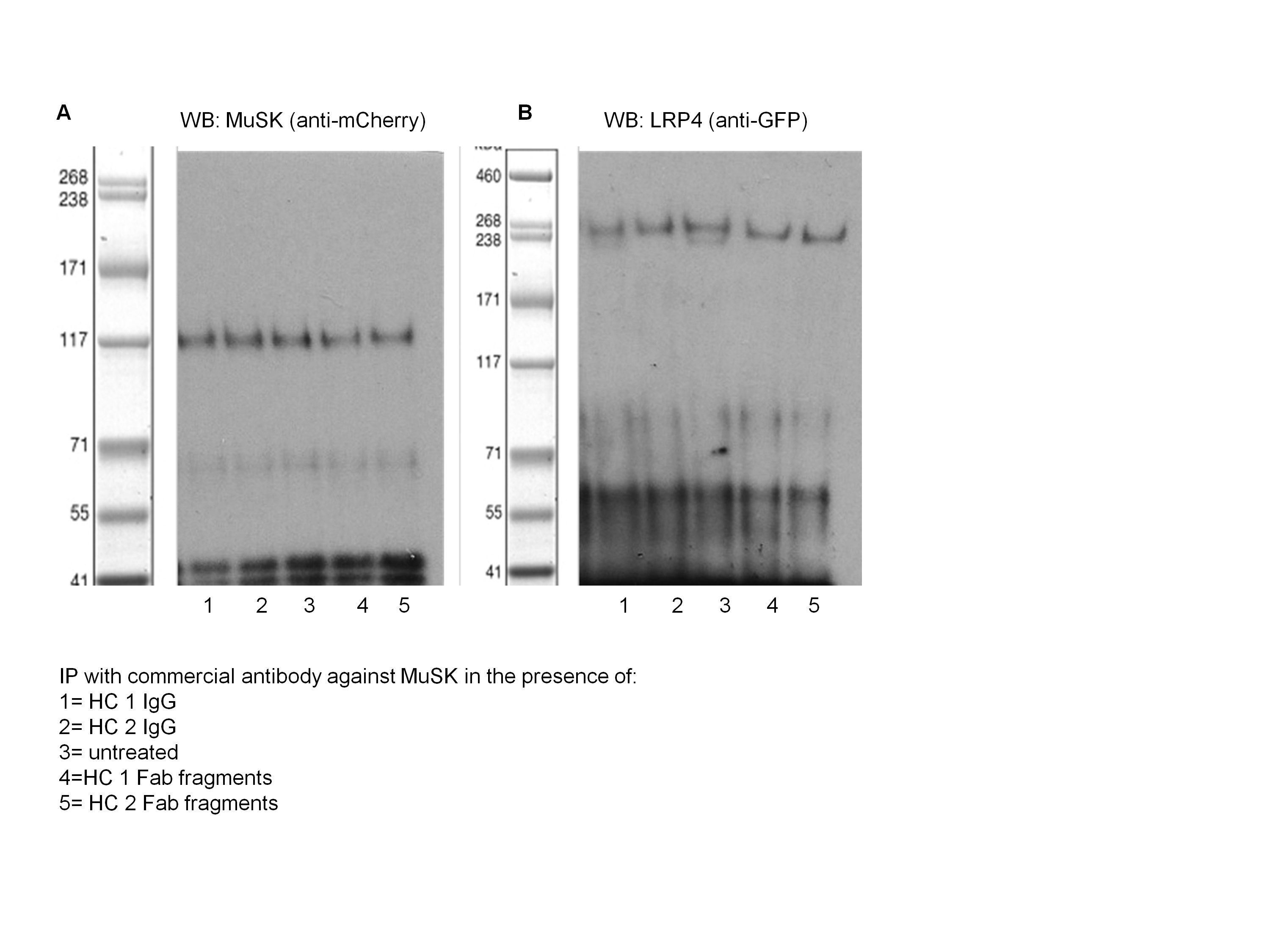

Supplement: Figure S6 — Healthy control IgG and healthy control Fab fragments do not interfere with MuSK-LRP4 binding. MuSK-mCherry was immuno-precipitated from the cell surface of HEK293 cells transfected with MuSK-mCherry and LRP4-EGFP using a goat anti-MuSK antibody alone, or in the presence of healthy control IgG (1,2) or healthy control Fab fragments (4,5) at volumes equal to those of patient samples used in experiments. (A) Western blot using an anti-mCherry antibody to detect MuSK-mCherry, and (B) Western blot with anti-GFP to detect LRP4-EGFP. Similar amounts of MuSK-mCherry and LRP4-EGFP were precipitated in all samples. (TIF) [file pone.0080695.s006.tif]

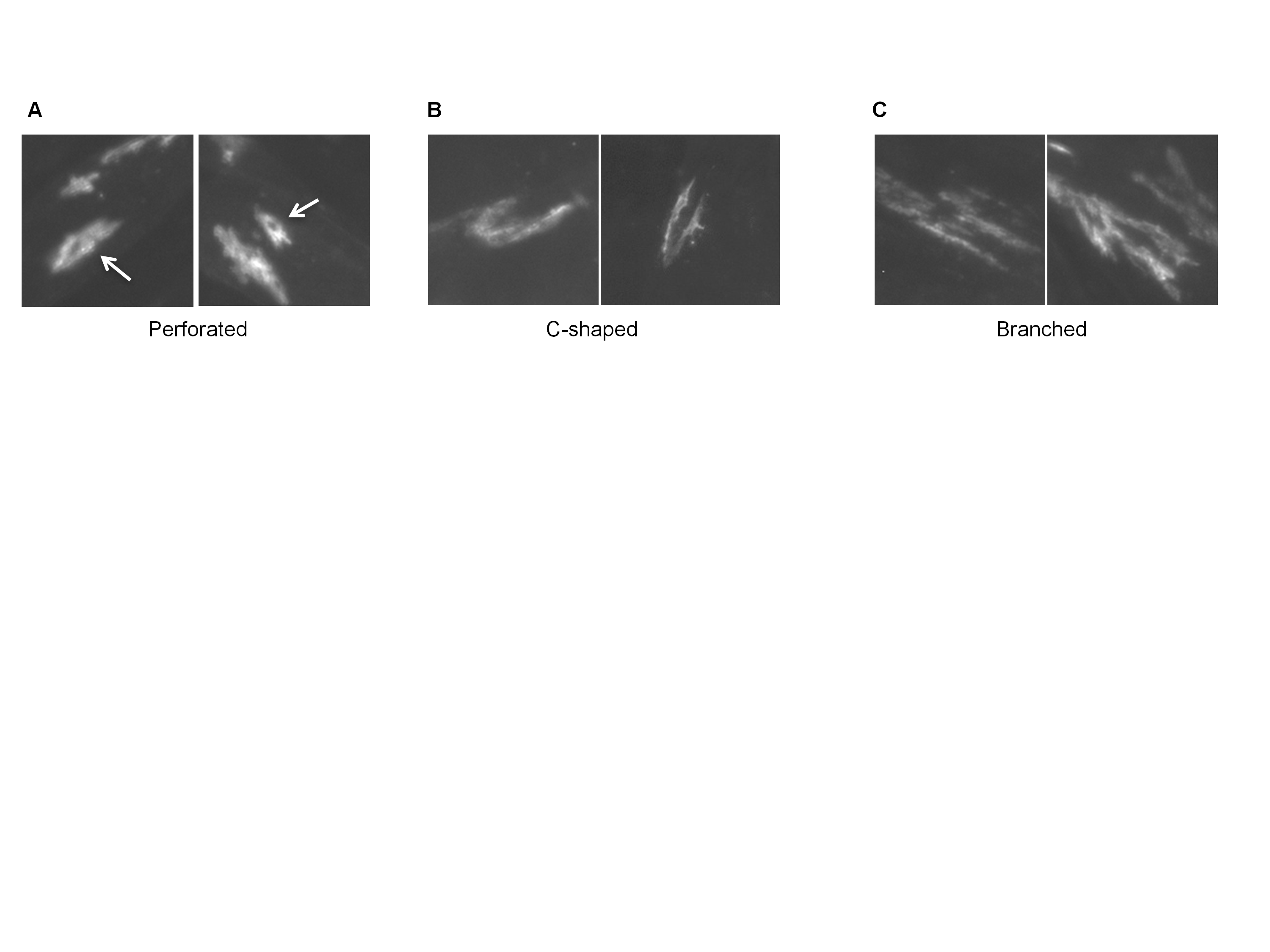

Supplement: Figure S7 — Examples of complex AChR clusters that were induced by overexpressing Dok7 in C2C12 myotubes and stained using Alexa Fluor 594-conjugated α-bungarotoxin. (A) Arrows indicate perforated clusters. (B) c-shaped clusters. (C) Branched clusters. (TIF) [file pone.0080695.s007.tif]

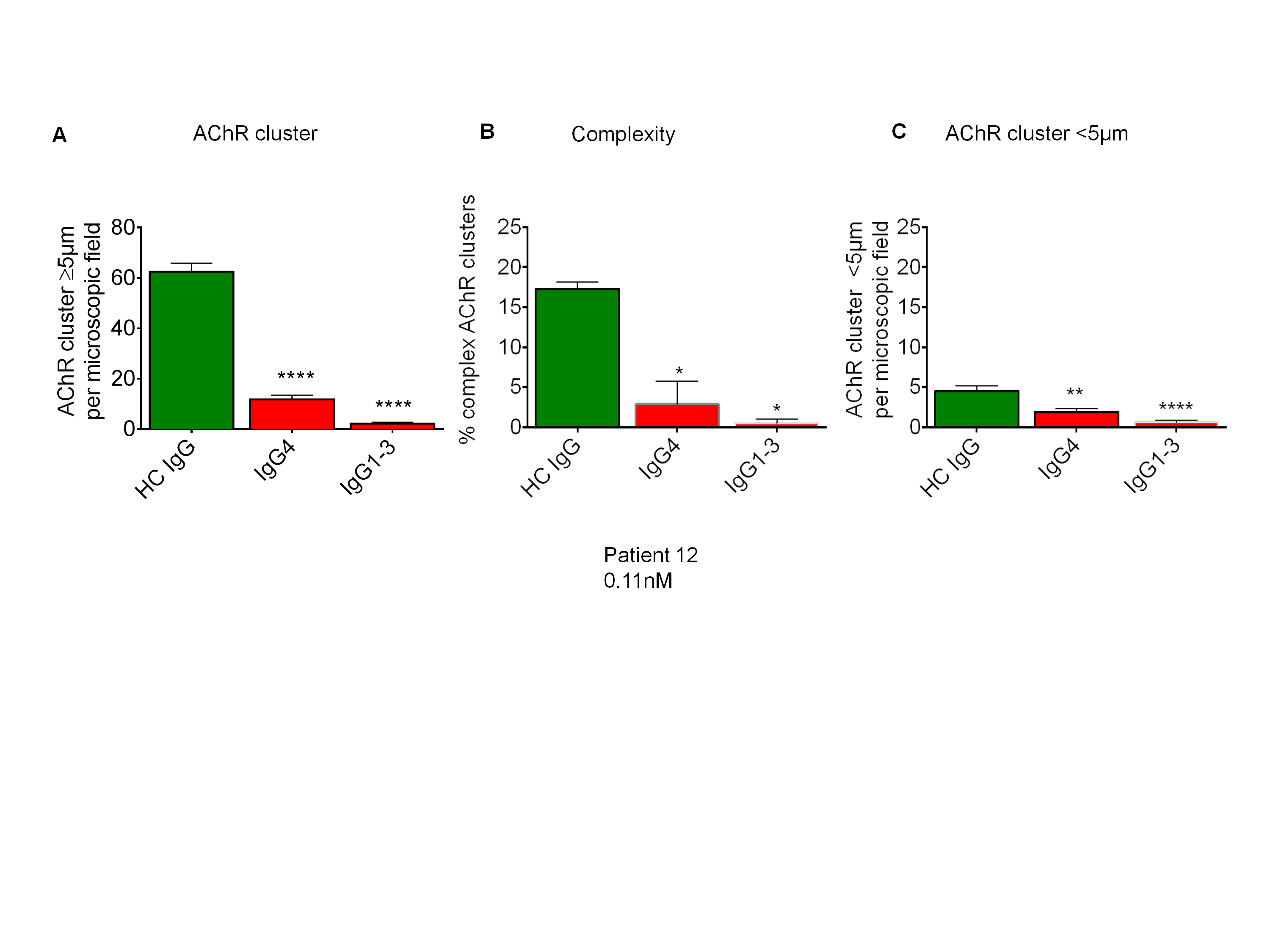

Supplement: Figure S8 — MuSK-MG patient IgG1-3 and IgG4 disrupt Dok7-induced AChR clustering on C2C12 myotubes. Myotubes were incubated overnight with 0.11nM MuSK specific IgG1-3 or IgG4 from patient 12. Results are the average of two experiments. (A) Number of AChR clusters ≥5μm was quantitated. (B) The proportion of complex AChR clusters (perforated, c-shaped and branched - see Figure S7) was calculated. (C) Number of AChR clusters <5µm was measured. One way ANOVA (p<0.0001) followed by Bonferroni post test. * p≤0.05, ** p≤0.01, **** p≤0.0001. (TIF) [file pone.0080695.s008.tif]
